# Supplementary material for: Tuesday's Teaching Tips—Evaluation and Feedback: A Spaced Education Strategy for Faculty Development
Source: MedEdPORTAL. 2022 Nov 22;18:11281. doi: 10.15766/mep_2374-8265.11281 (PMC9678823; doi:10.15766/mep_2374-8265.11281)

## Slide 1
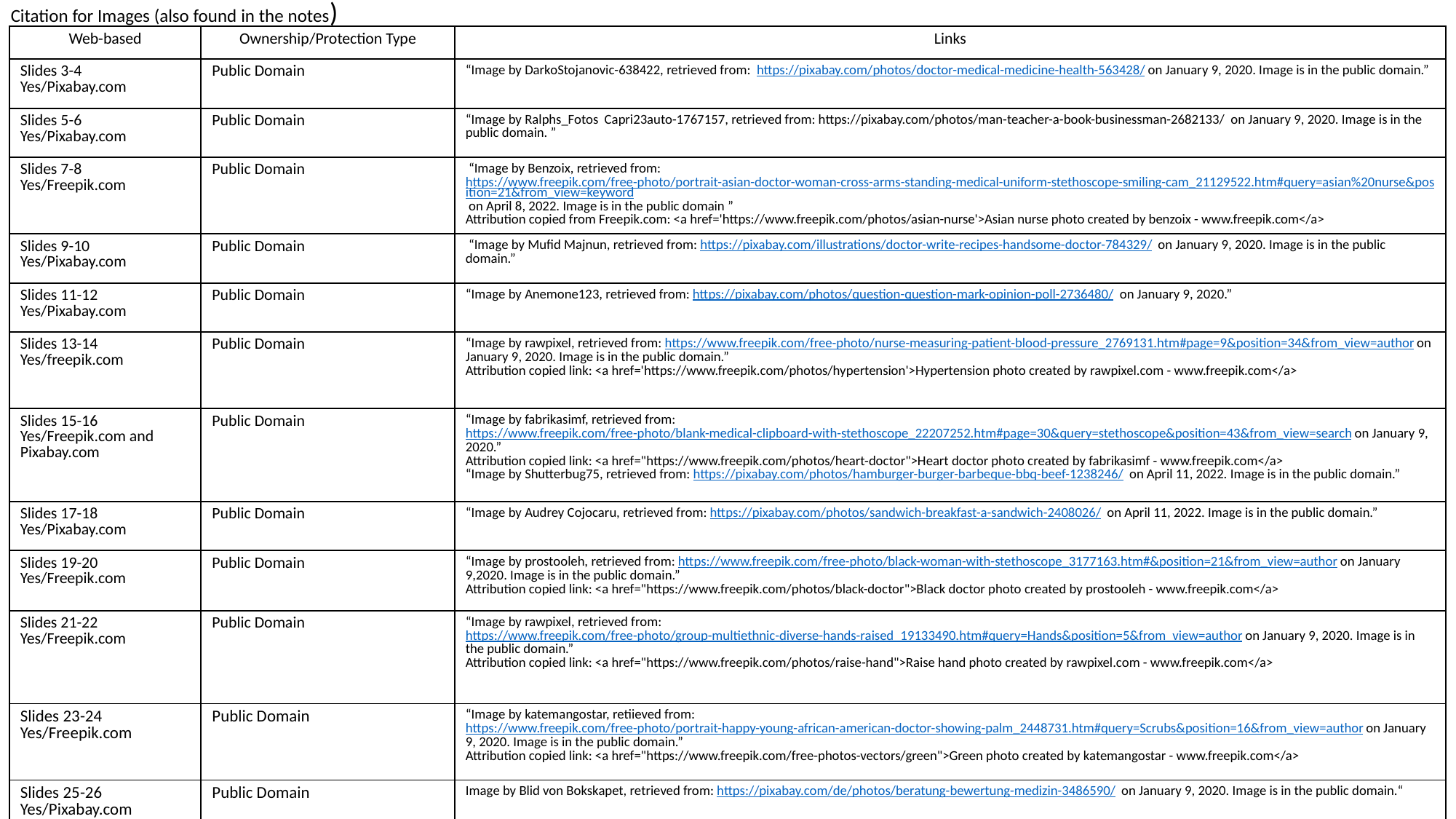

Citation for Images (also found in the notes)
| Web-based | Ownership/Protection Type | Links |
| --- | --- | --- |
| Slides 3-4 Yes/Pixabay.com | Public Domain | “Image by DarkoStojanovic-638422, retrieved from: https://pixabay.com/photos/doctor-medical-medicine-health-563428/ on January 9, 2020. Image is in the public domain.” |
| Slides 5-6 Yes/Pixabay.com | Public Domain | “Image by Ralphs\_Fotos Capri23auto-1767157, retrieved from: https://pixabay.com/photos/man-teacher-a-book-businessman-2682133/ on January 9, 2020. Image is in the public domain. ” |
| Slides 7-8 Yes/Freepik.com | Public Domain | “Image by Benzoix, retrieved from: https://www.freepik.com/free-photo/portrait-asian-doctor-woman-cross-arms-standing-medical-uniform-stethoscope-smiling-cam\_21129522.htm#query=asian%20nurse&position=21&from\_view=keyword on April 8, 2022. Image is in the public domain ” Attribution copied from Freepik.com: <a href='https://www.freepik.com/photos/asian-nurse'>Asian nurse photo created by benzoix - www.freepik.com</a> |
| Slides 9-10 Yes/Pixabay.com | Public Domain | “Image by Mufid Majnun, retrieved from: https://pixabay.com/illustrations/doctor-write-recipes-handsome-doctor-784329/ on January 9, 2020. Image is in the public domain.” |
| Slides 11-12 Yes/Pixabay.com | Public Domain | “Image by Anemone123, retrieved from: https://pixabay.com/photos/question-question-mark-opinion-poll-2736480/ on January 9, 2020.” |
| Slides 13-14 Yes/freepik.com | Public Domain | “Image by rawpixel, retrieved from: https://www.freepik.com/free-photo/nurse-measuring-patient-blood-pressure\_2769131.htm#page=9&position=34&from\_view=author on January 9, 2020. Image is in the public domain.” Attribution copied link: <a href='https://www.freepik.com/photos/hypertension'>Hypertension photo created by rawpixel.com - www.freepik.com</a> |
| Slides 15-16 Yes/Freepik.com and Pixabay.com | Public Domain | “Image by fabrikasimf, retrieved from: https://www.freepik.com/free-photo/blank-medical-clipboard-with-stethoscope\_22207252.htm#page=30&query=stethoscope&position=43&from\_view=search on January 9, 2020.” Attribution copied link: <a href="https://www.freepik.com/photos/heart-doctor">Heart doctor photo created by fabrikasimf - www.freepik.com</a> “Image by Shutterbug75, retrieved from: https://pixabay.com/photos/hamburger-burger-barbeque-bbq-beef-1238246/ on April 11, 2022. Image is in the public domain.” |
| Slides 17-18 Yes/Pixabay.com | Public Domain | “Image by Audrey Cojocaru, retrieved from: https://pixabay.com/photos/sandwich-breakfast-a-sandwich-2408026/ on April 11, 2022. Image is in the public domain.” |
| Slides 19-20 Yes/Freepik.com | Public Domain | “Image by prostooleh, retrieved from: https://www.freepik.com/free-photo/black-woman-with-stethoscope\_3177163.htm#&position=21&from\_view=author on January 9,2020. Image is in the public domain.” Attribution copied link: <a href="https://www.freepik.com/photos/black-doctor">Black doctor photo created by prostooleh - www.freepik.com</a> |
| Slides 21-22 Yes/Freepik.com | Public Domain | “Image by rawpixel, retrieved from: https://www.freepik.com/free-photo/group-multiethnic-diverse-hands-raised\_19133490.htm#query=Hands&position=5&from\_view=author on January 9, 2020. Image is in the public domain.” Attribution copied link: <a href="https://www.freepik.com/photos/raise-hand">Raise hand photo created by rawpixel.com - www.freepik.com</a> |
| Slides 23-24 Yes/Freepik.com | Public Domain | “Image by katemangostar, retiieved from: https://www.freepik.com/free-photo/portrait-happy-young-african-american-doctor-showing-palm\_2448731.htm#query=Scrubs&position=16&from\_view=author on January 9, 2020. Image is in the public domain.” Attribution copied link: <a href="https://www.freepik.com/free-photos-vectors/green">Green photo created by katemangostar - www.freepik.com</a> |
| Slides 25-26 Yes/Pixabay.com | Public Domain | Image by Blid von Bokskapet, retrieved from: https://pixabay.com/de/photos/beratung-bewertung-medizin-3486590/ on January 9, 2020. Image is in the public domain.“ |
| Slides 27-28 Yes/Pixabay.com | Public Domain | “Image by DarkoStojanovic, retrieved from: https://pixabay.com/photos/medic-hospital-laboratory-medical-563425/ on January 9, 2020. Image is in the public domain.” |

## Slide 2
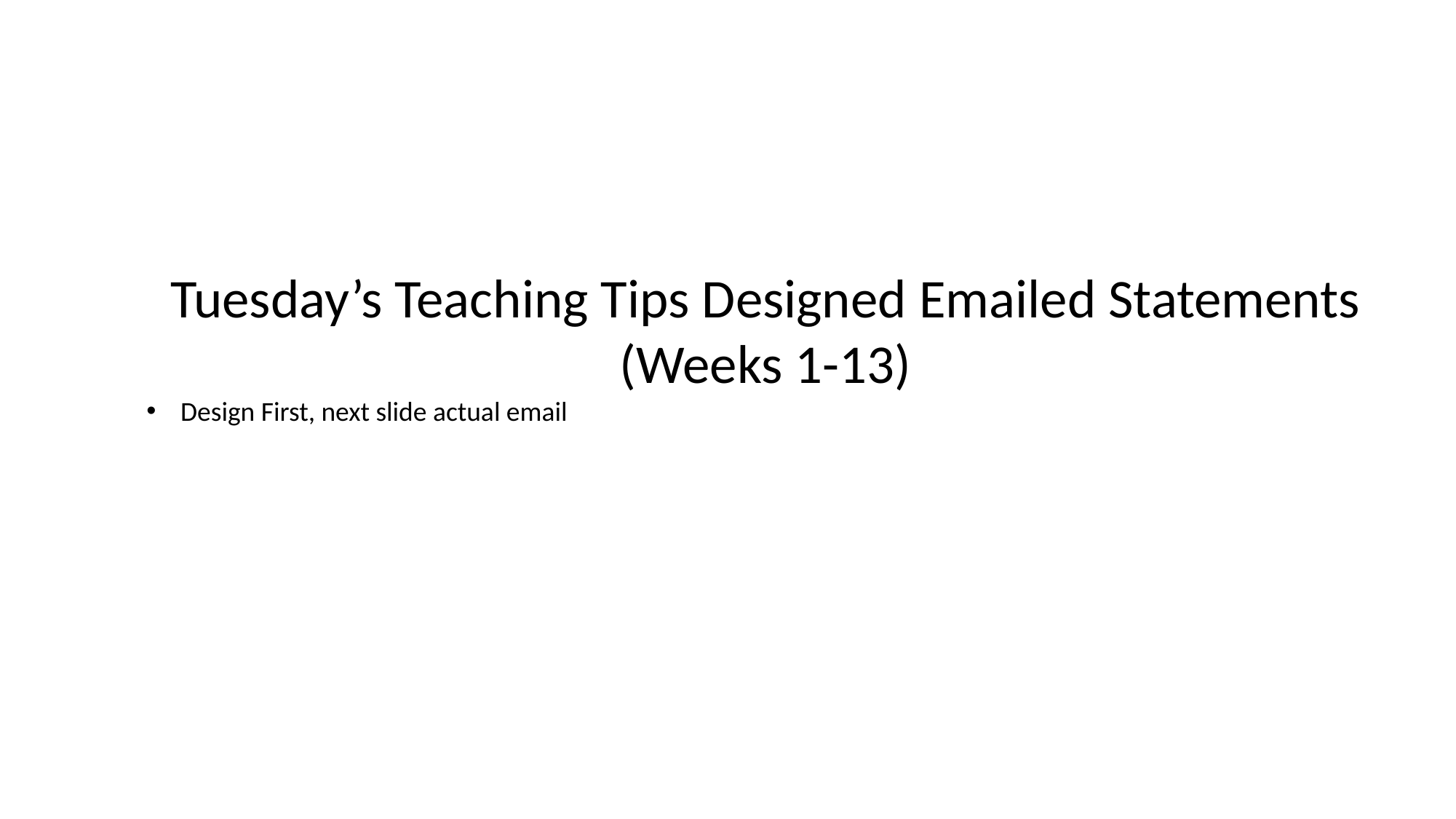

Tuesday’s Teaching Tips Designed Emailed Statements (Weeks 1-13)
Design First, next slide actual email

## Slide 3
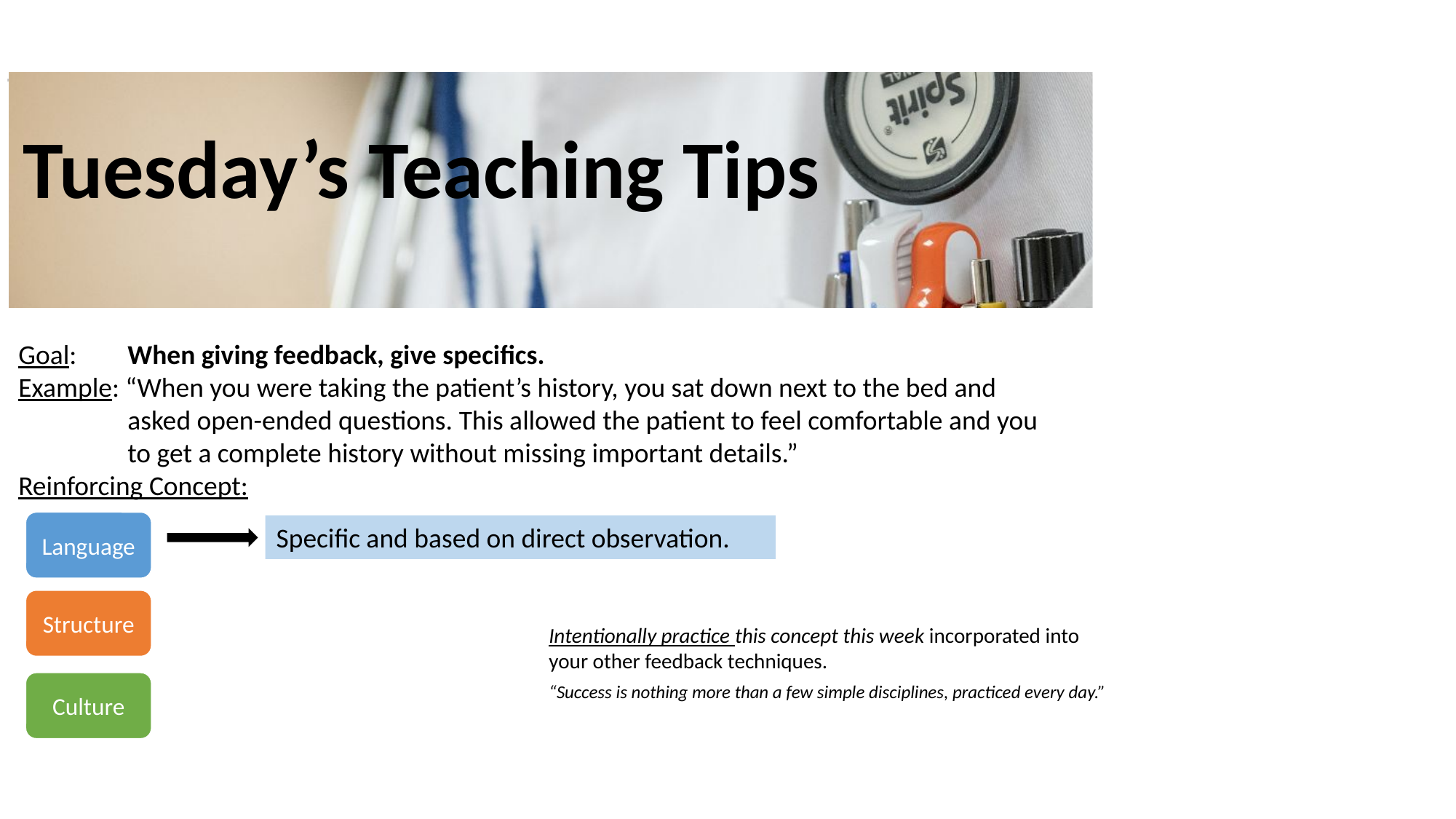

Tuesday’s Teaching Tips
Goal: 	When giving feedback, give specifics.
Example: “When you were taking the patient’s history, you sat down next to the bed and 	asked open-ended questions. This allowed the patient to feel comfortable and you 	to get a complete history without missing important details.”
Reinforcing Concept:
Language
Specific and based on direct observation.
Structure
Intentionally practice this concept this week incorporated into your other feedback techniques.
“Success is nothing more than a few simple disciplines, practiced every day.”
Culture

## Slide 4
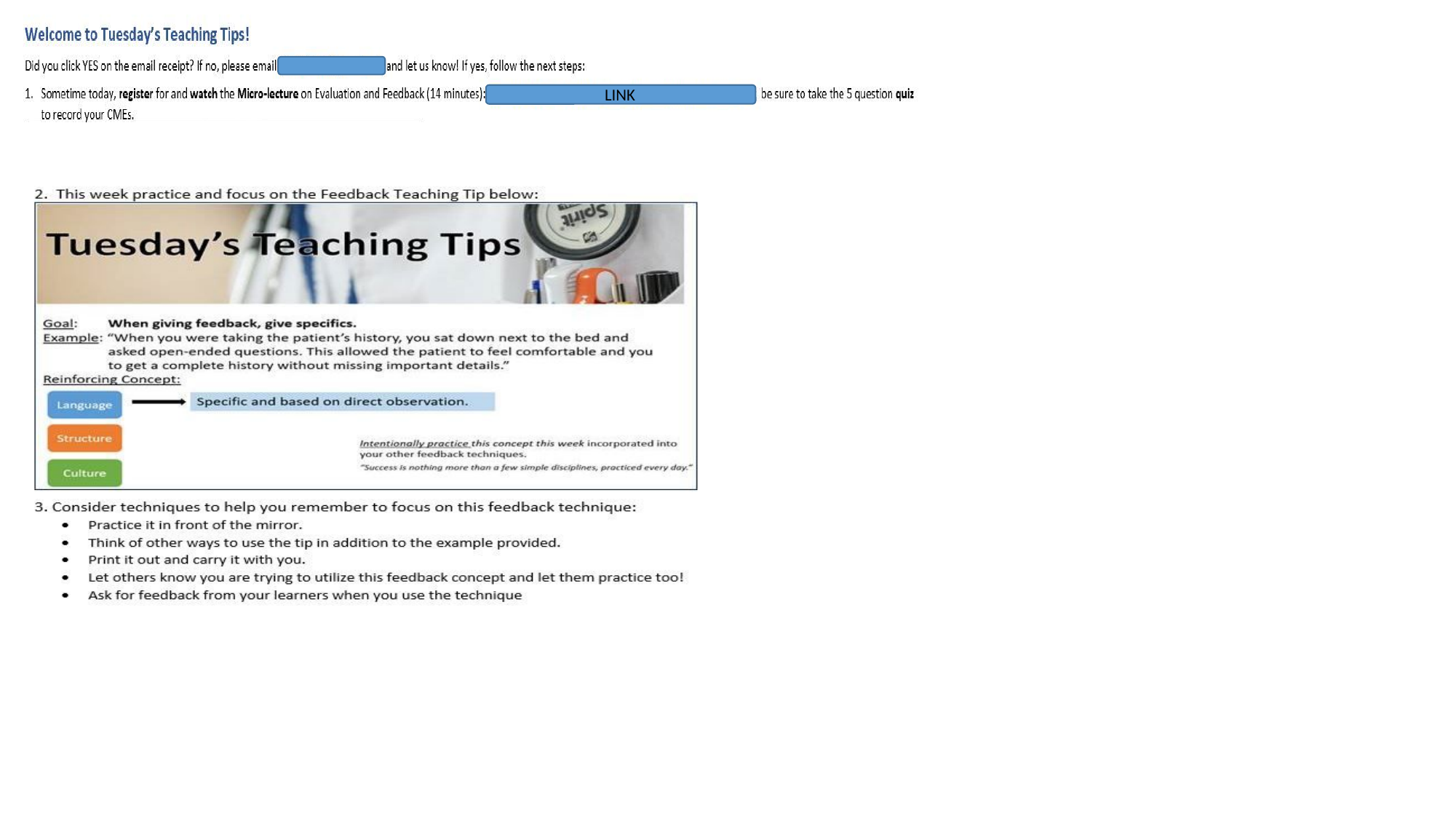

LINK

## Slide 5
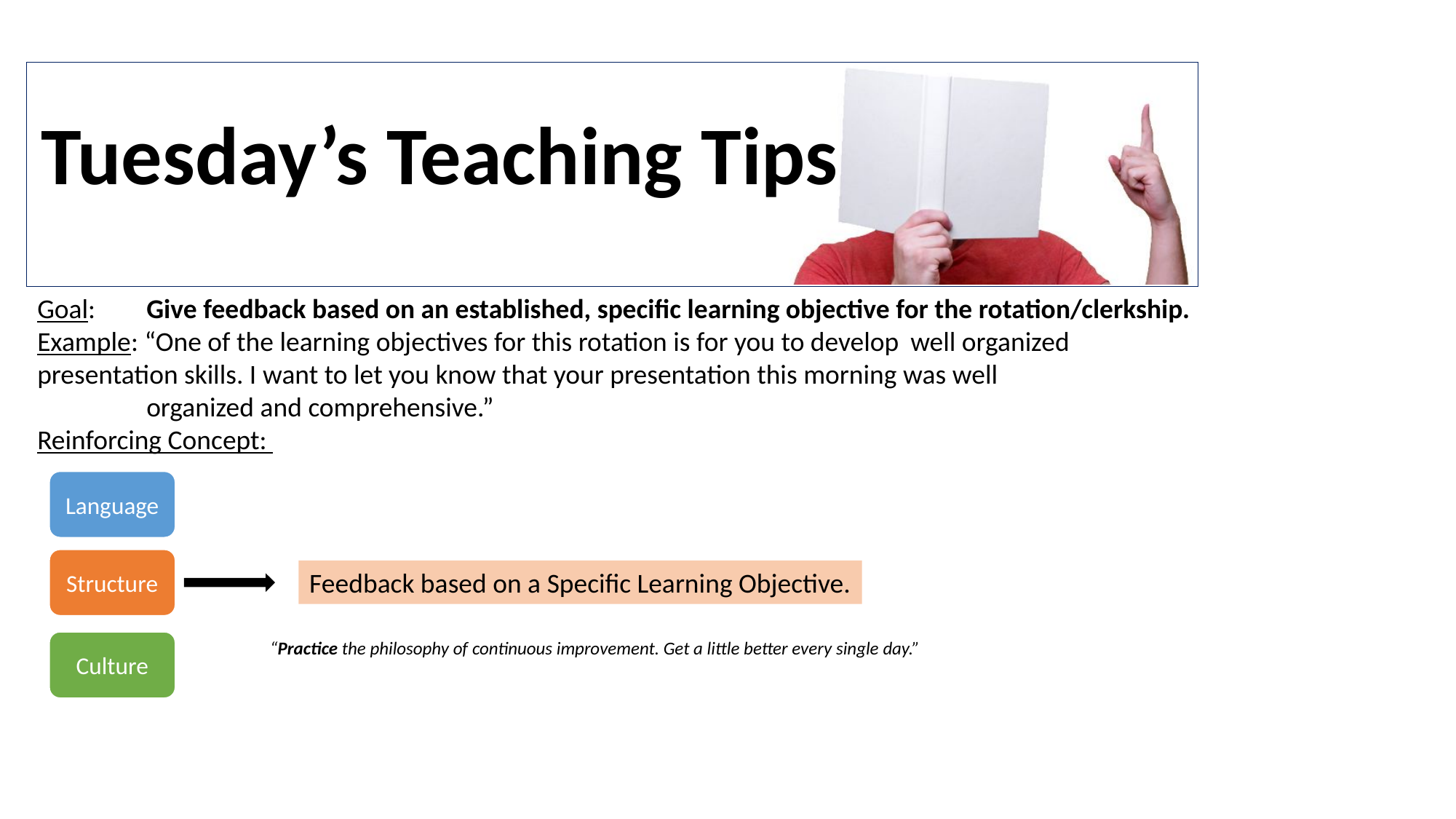

Tuesday’s Teaching Tips
Goal: 	Give feedback based on an established, specific learning objective for the rotation/clerkship.
Example: “One of the learning objectives for this rotation is for you to develop 	well organized 	presentation skills. I want to let you know that your presentation this morning was well
	organized and comprehensive.”
Reinforcing Concept:
Language
Structure
Feedback based on a Specific Learning Objective.
Culture
“Practice the philosophy of continuous improvement. Get a little better every single day.”

## Slide 6
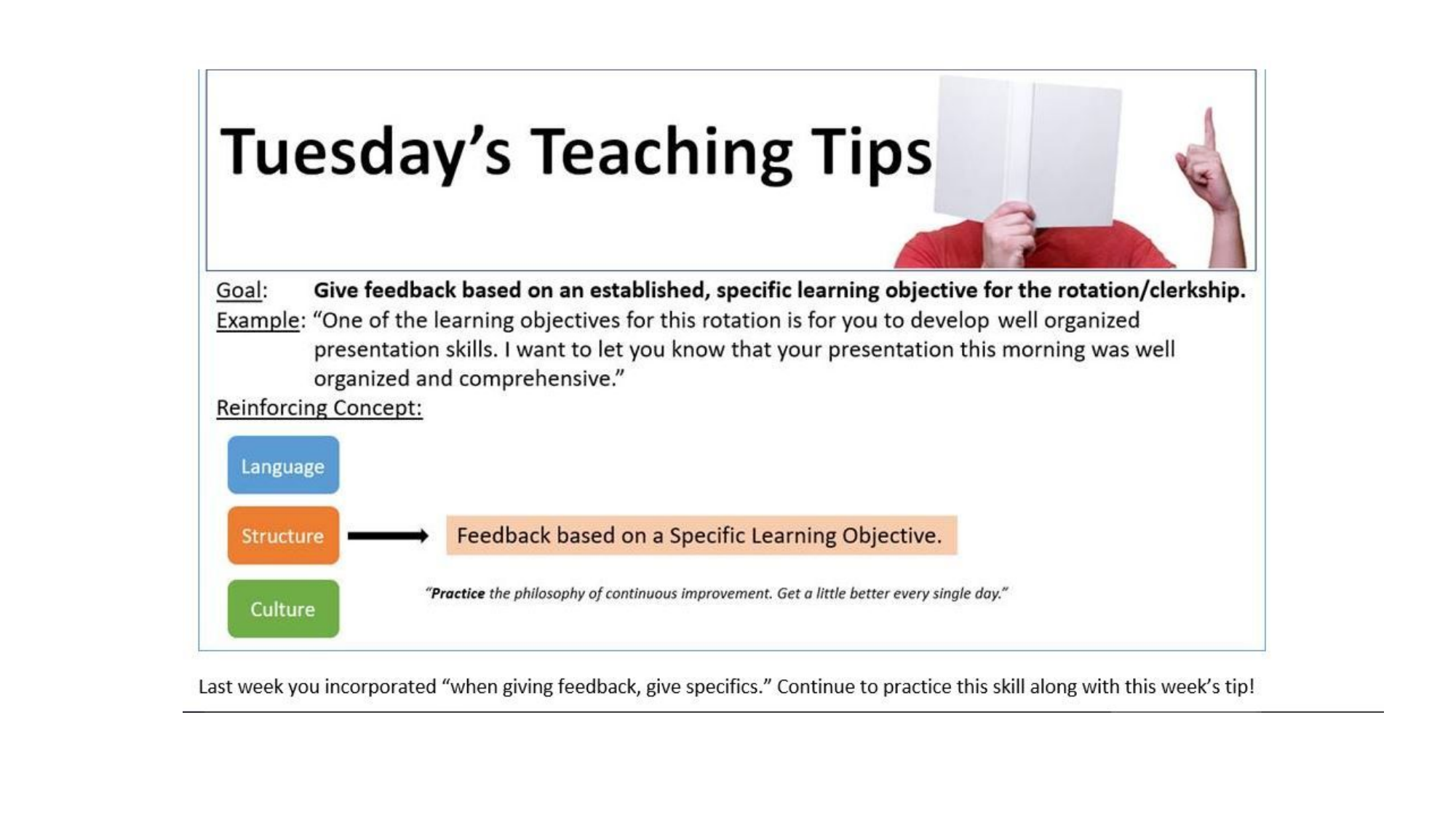

## Slide 7
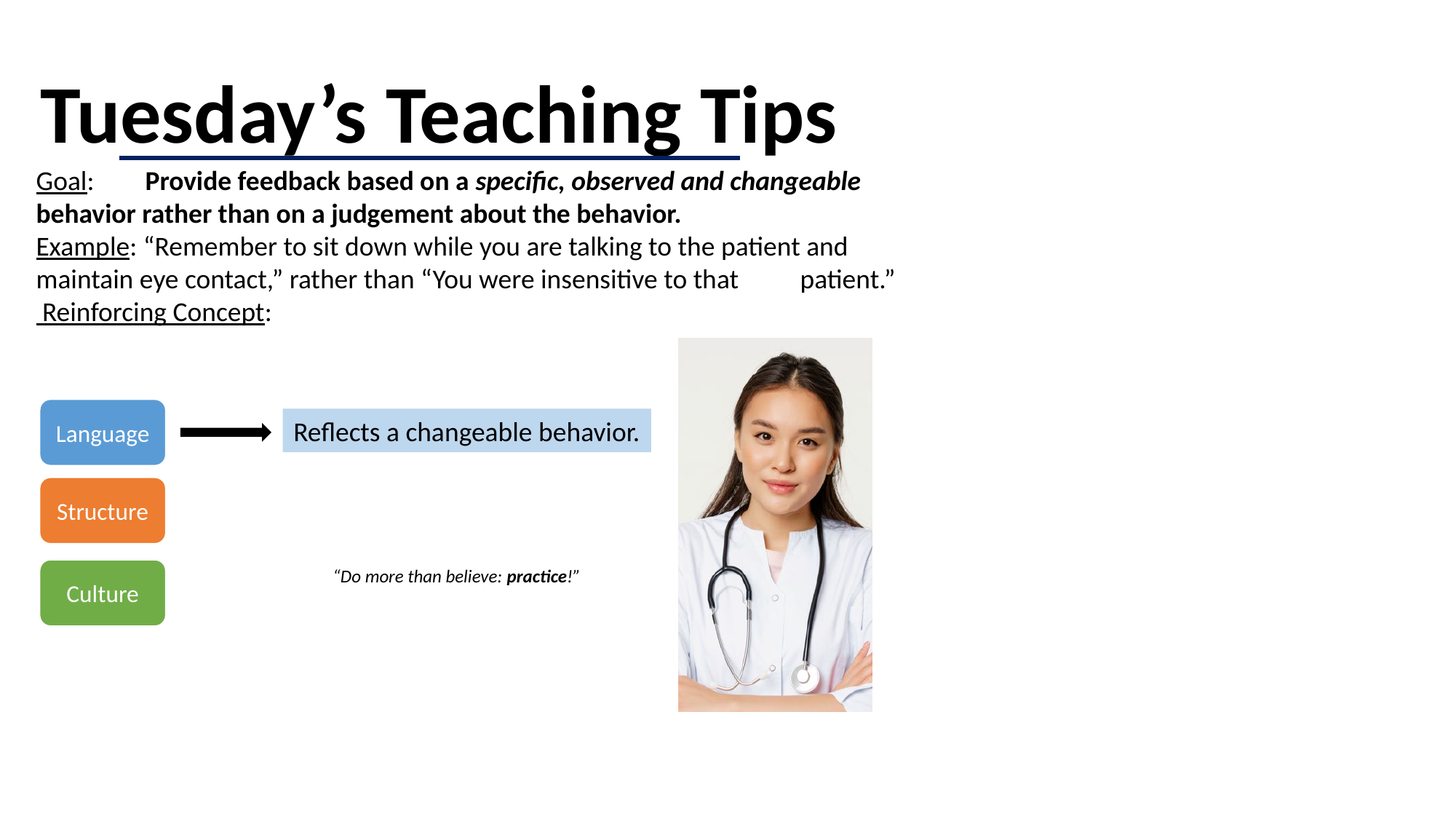

Tuesday’s Teaching Tips
Goal: 	Provide feedback based on a specific, observed and changeable 	behavior rather than on a judgement about the behavior.
Example: “Remember to sit down while you are talking to the patient and 	maintain eye contact,” rather than “You were insensitive to that 	patient.”
 Reinforcing Concept:
Language
Reflects a changeable behavior.
Structure
Culture
“Do more than believe: practice!”

## Slide 8
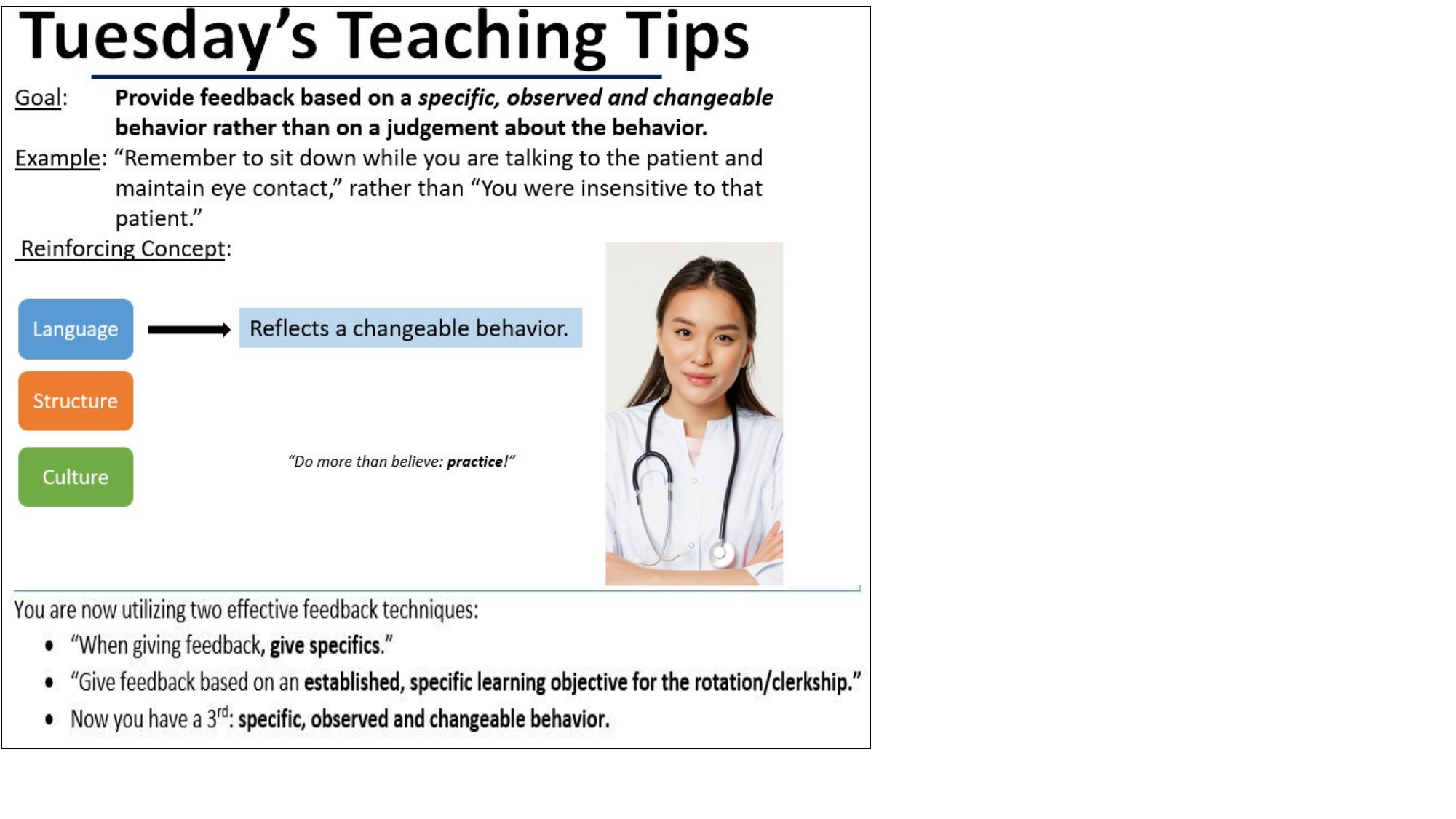

## Slide 9
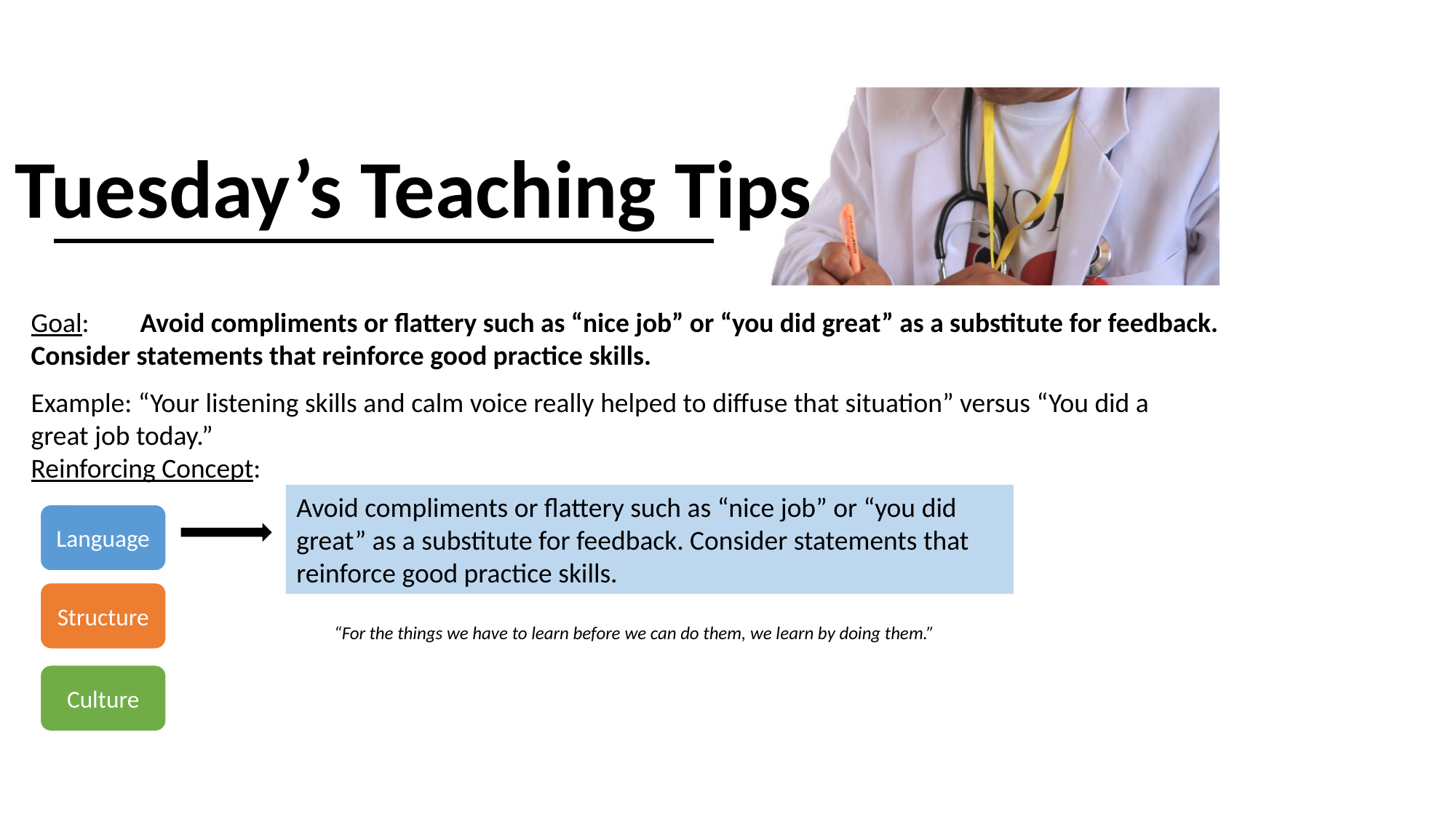

Tuesday’s Teaching Tips
Goal: 	Avoid compliments or flattery such as “nice job” or “you did great” as a substitute for feedback. 	Consider statements that reinforce good practice skills.
Example: “Your listening skills and calm voice really helped to diffuse that situation” versus “You did a 	great job today.”
Reinforcing Concept:
Avoid compliments or flattery such as “nice job” or “you did great” as a substitute for feedback. Consider statements that reinforce good practice skills.
Language
Structure
“For the things we have to learn before we can do them, we learn by doing them.”
Culture

## Slide 10
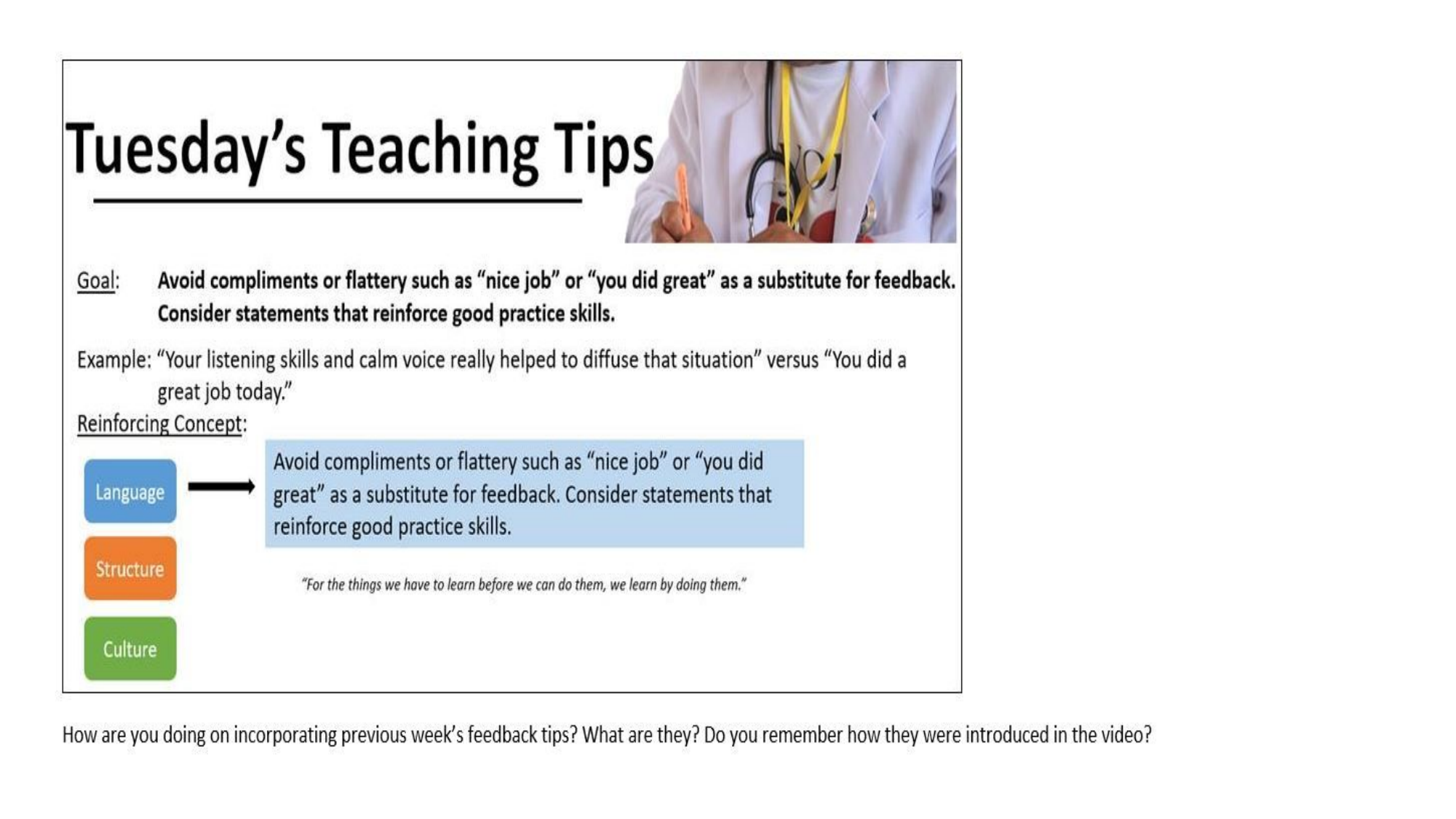

## Slide 11
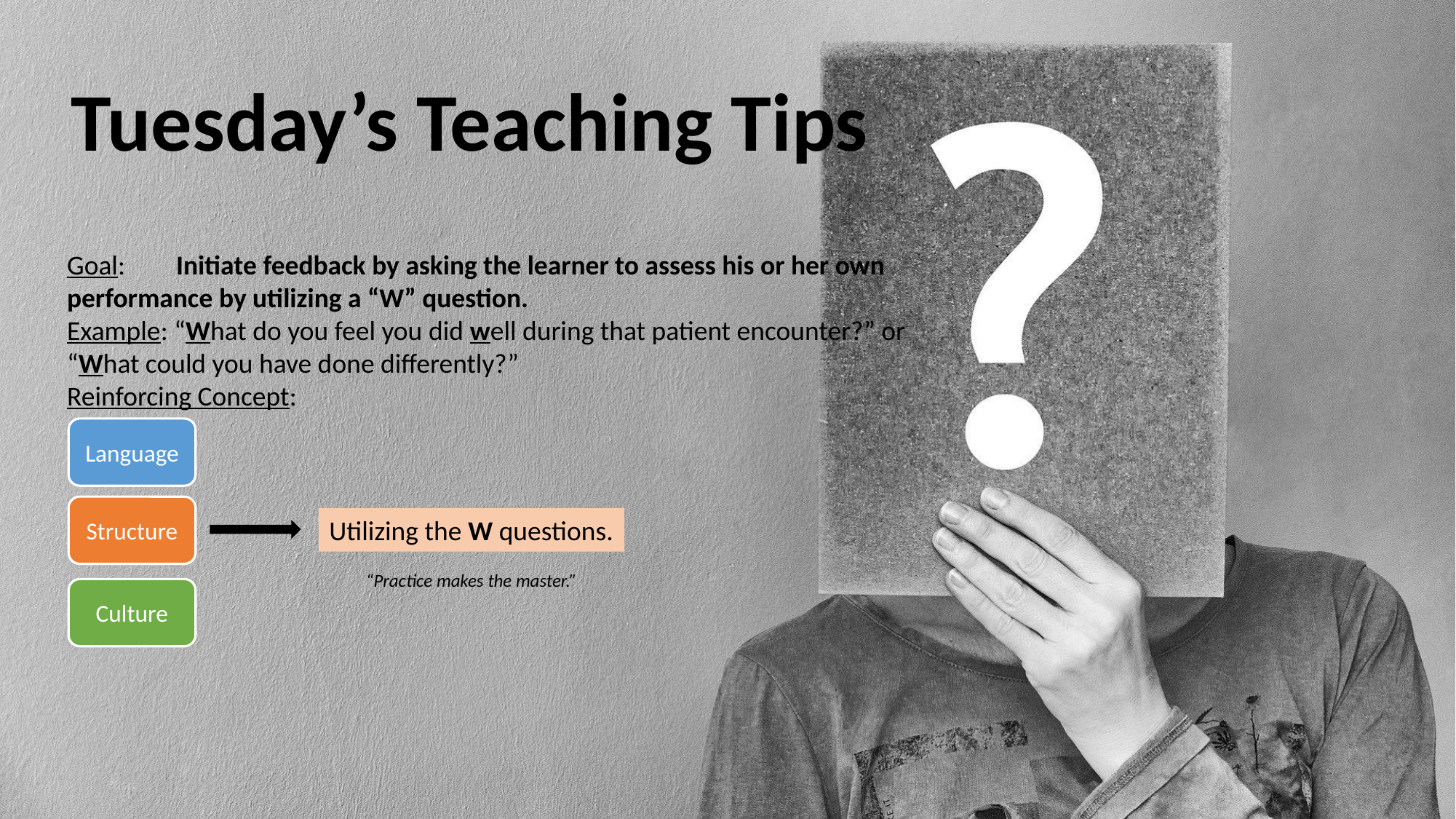

Tuesday’s Teaching Tips
Goal: 	Initiate feedback by asking the learner to assess his or her own 	performance by utilizing a “W” question.
Example: “What do you feel you did well during that patient encounter?” or 	“What could you have done differently?”
Reinforcing Concept:
Language
Structure
Utilizing the W questions.
“Practice makes the master.”
Culture

## Slide 12
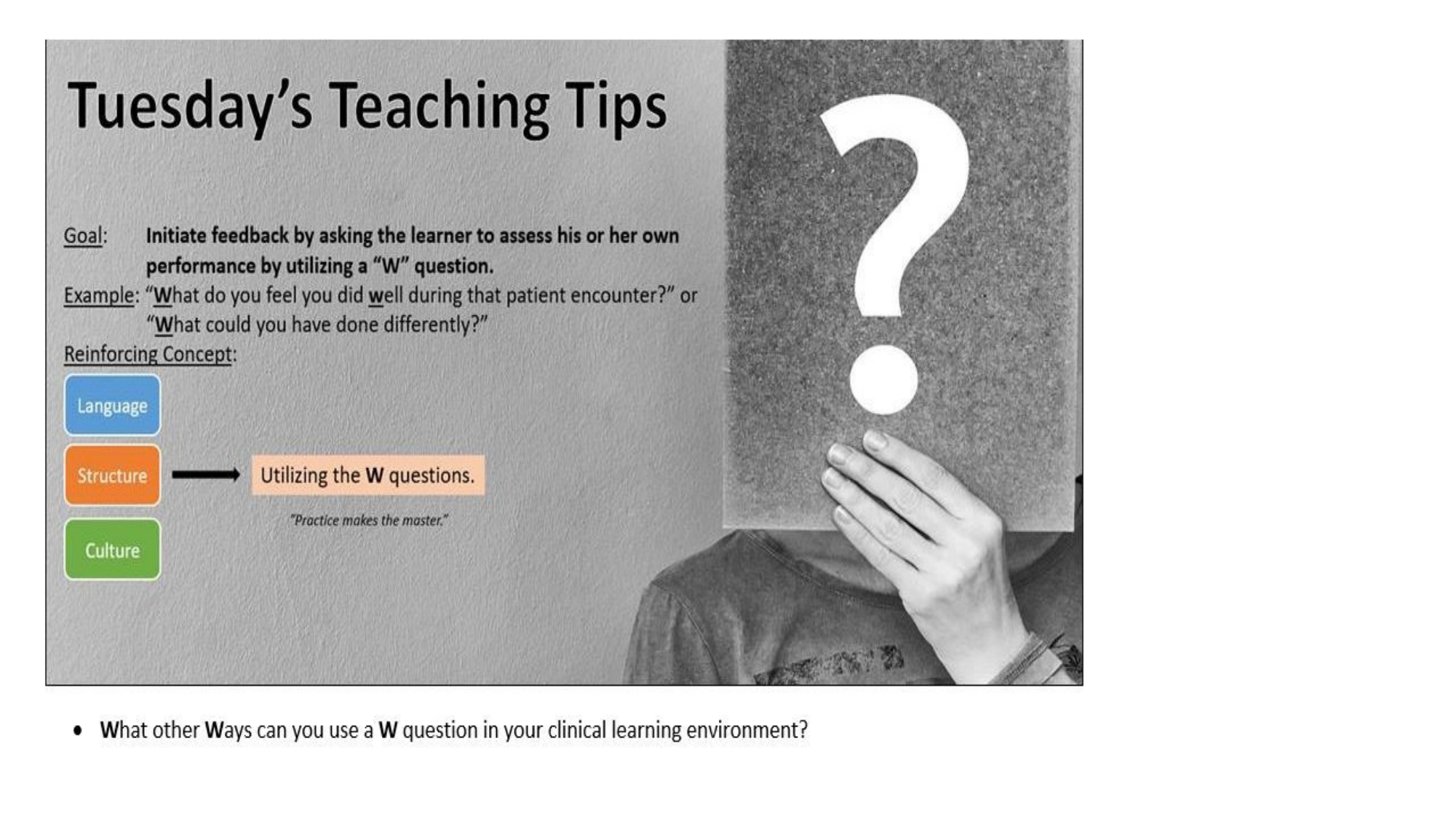

## Slide 13
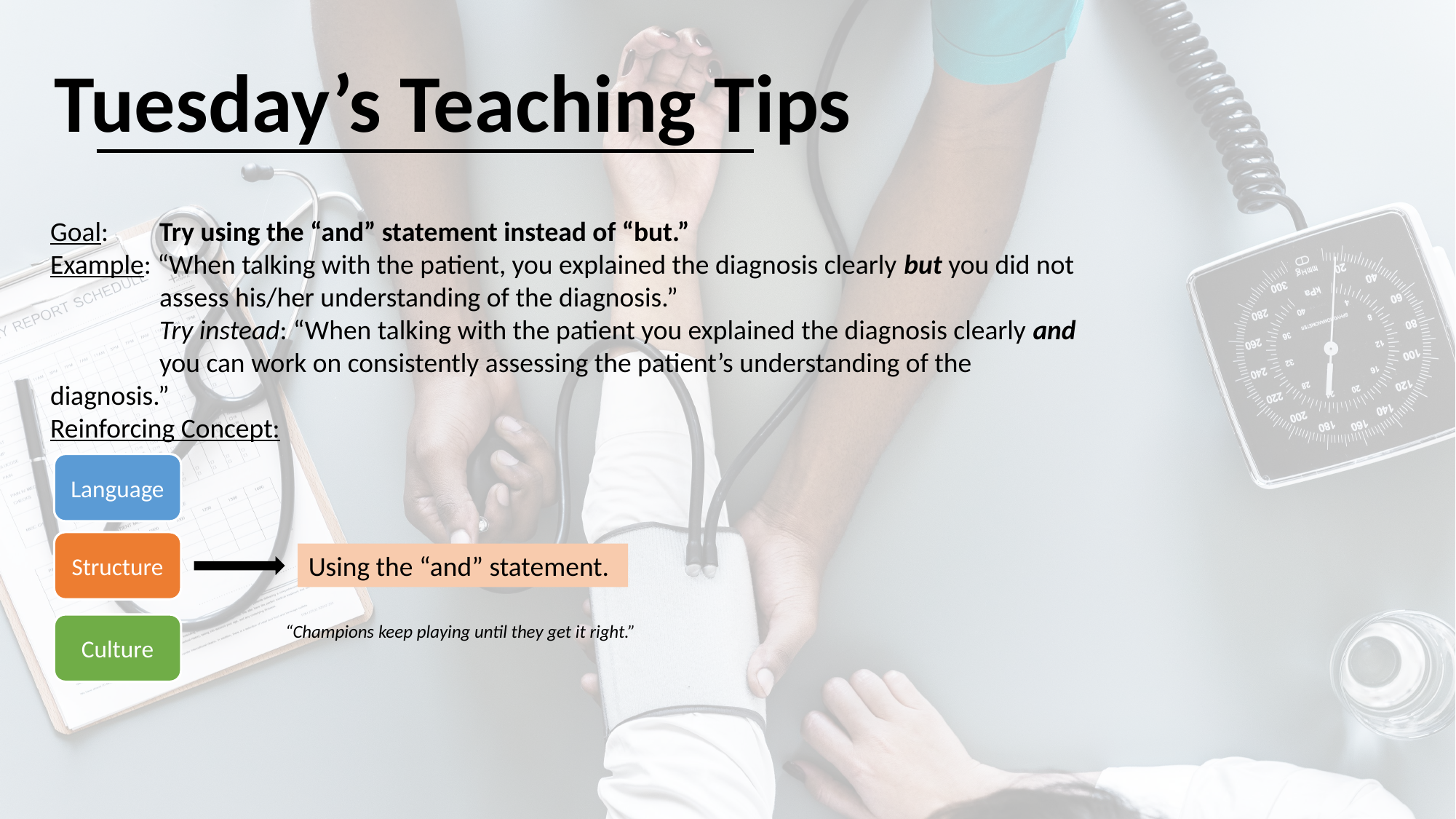

Tuesday’s Teaching Tips
Goal: 	Try using the “and” statement instead of “but.”
Example: “When talking with the patient, you explained the diagnosis clearly but you did not 	assess his/her understanding of the diagnosis.”
	Try instead: “When talking with the patient you explained the diagnosis clearly and 	you can work on consistently assessing the patient’s understanding of the 	diagnosis.”
Reinforcing Concept:
Language
Structure
Using the “and” statement.
“Champions keep playing until they get it right.”
Culture

## Slide 14
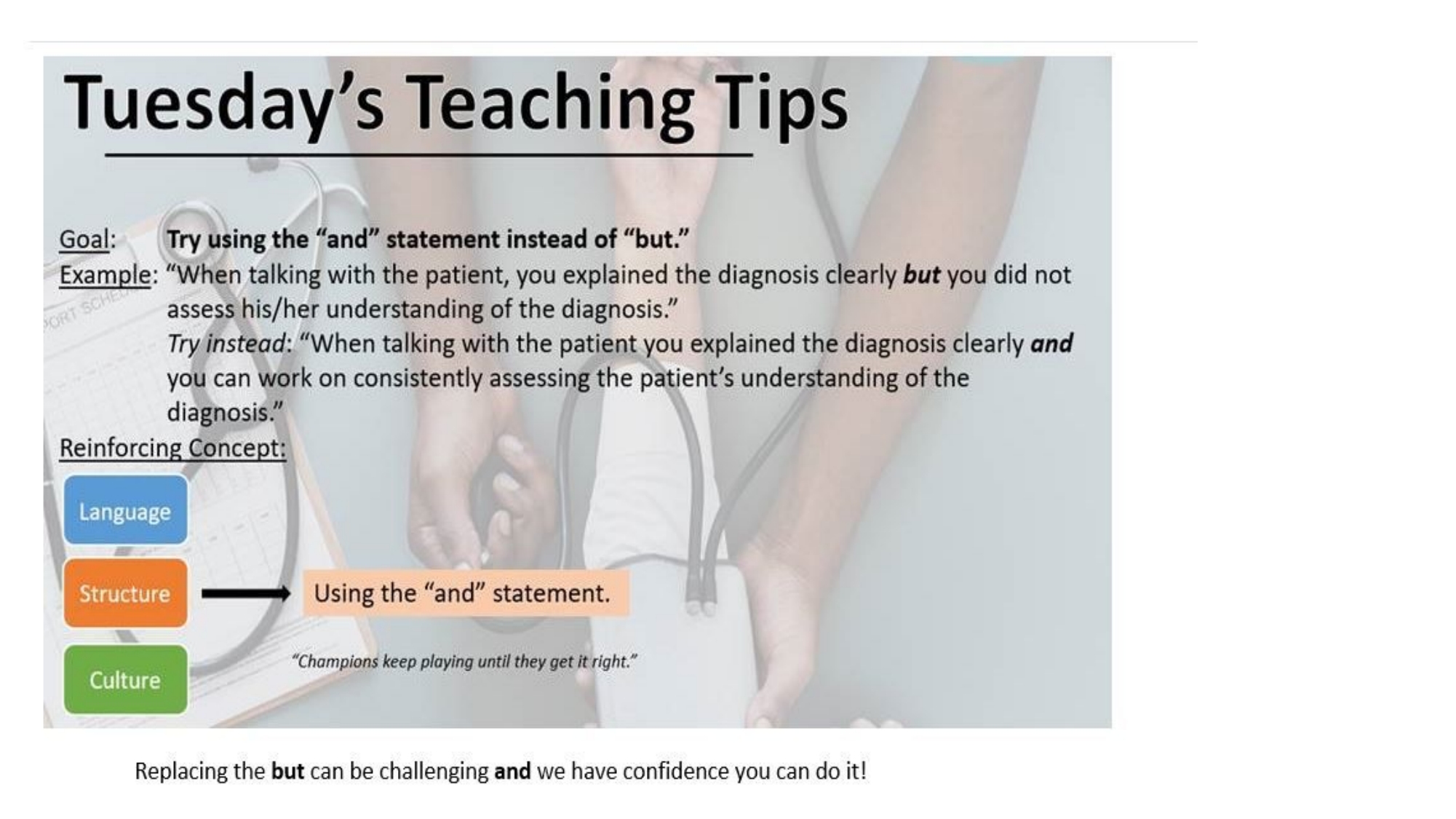

## Slide 15
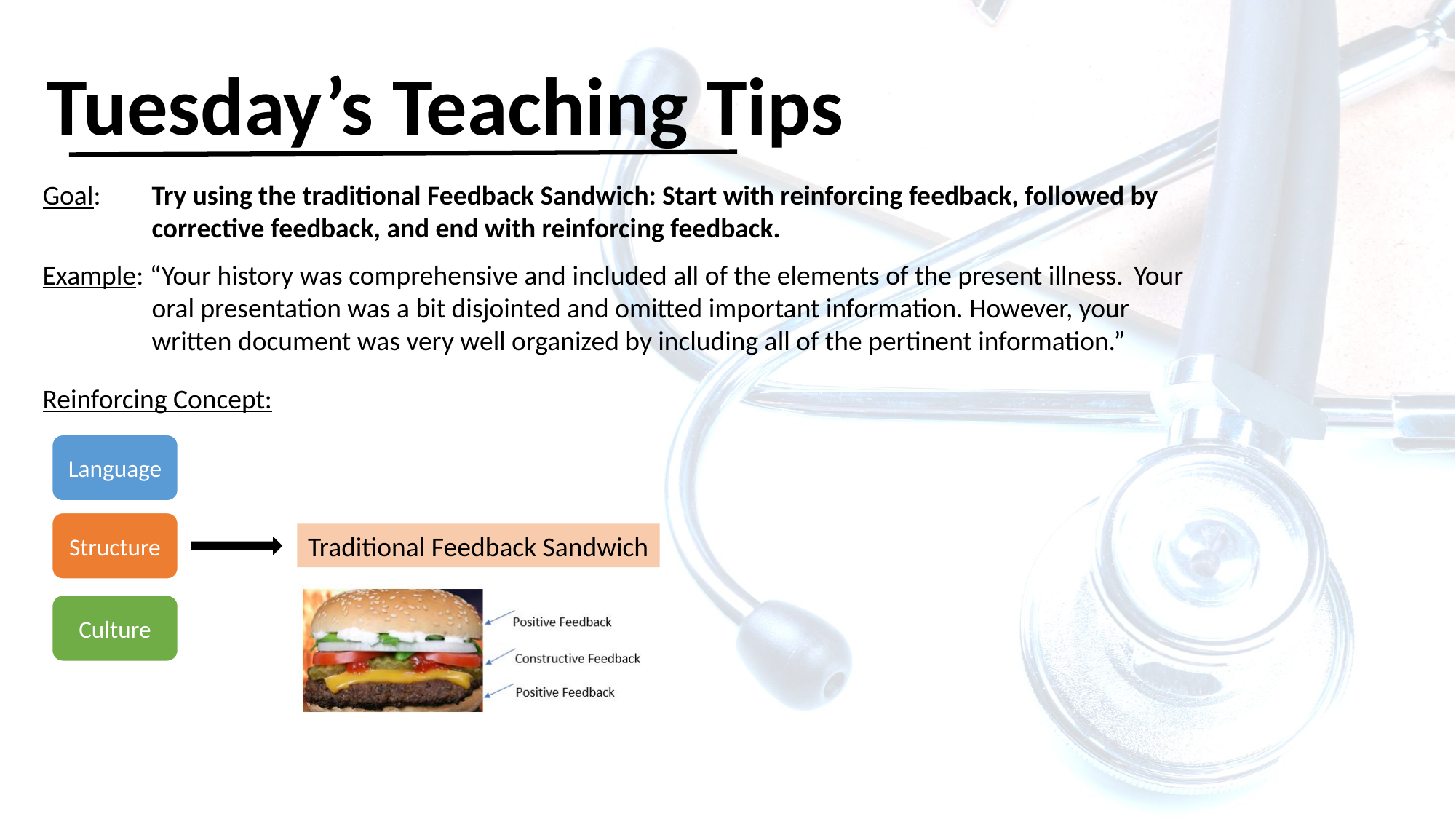

Tuesday’s Teaching Tips
Goal: 	Try using the traditional Feedback Sandwich: Start with reinforcing feedback, followed by 	corrective feedback, and end with reinforcing feedback.
Example: “Your history was comprehensive and included all of the elements of the present illness. 	Your 	oral presentation was a bit disjointed and omitted important information. However, your 	written document was very well organized by including all of the pertinent information.”
Reinforcing Concept:
Language
Structure
Traditional Feedback Sandwich
Culture

## Slide 16
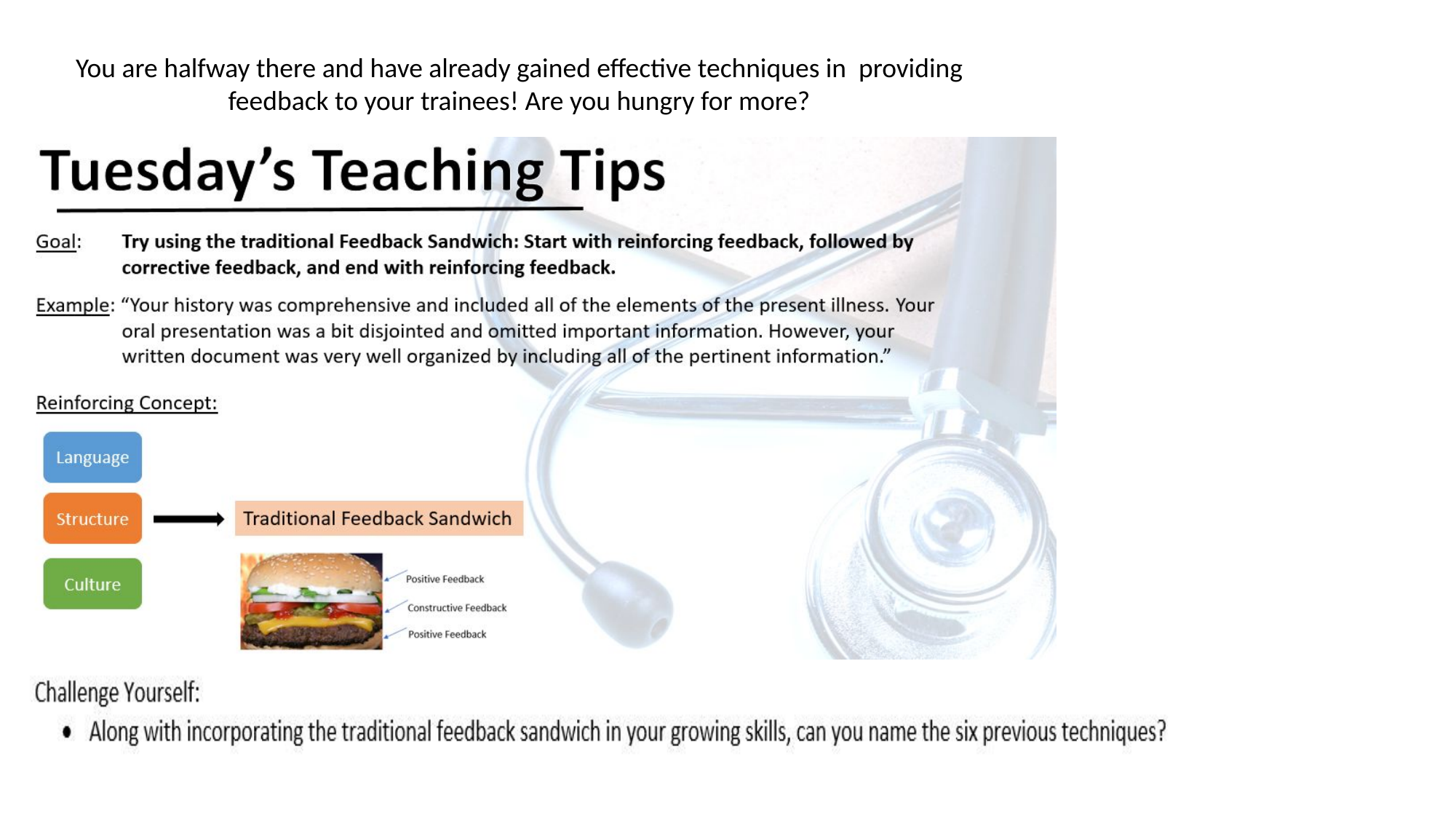

You are halfway there and have already gained effective techniques in providing feedback to your trainees! Are you hungry for more?

## Slide 17
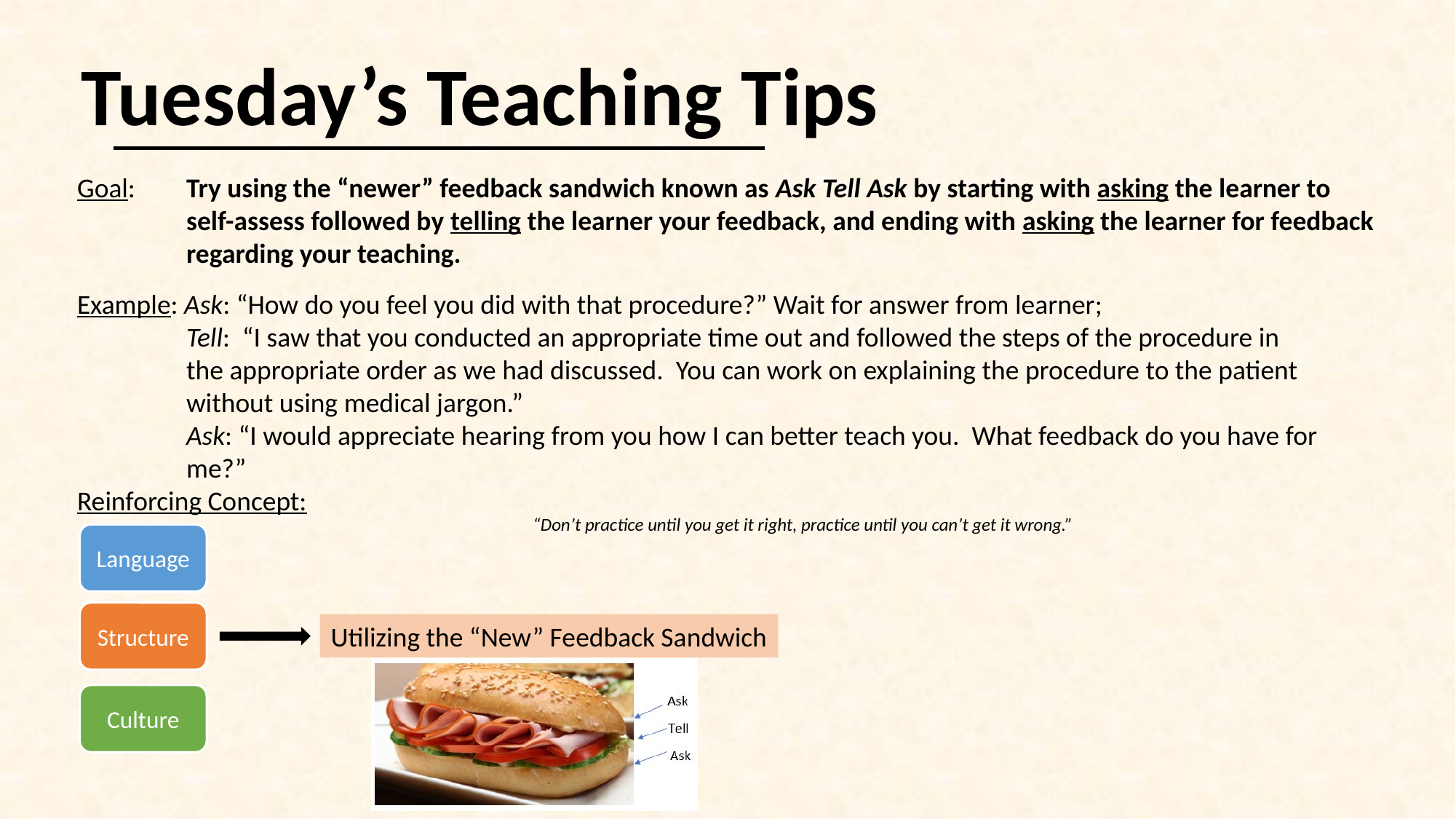

Tuesday’s Teaching Tips
Goal: 	Try using the “newer” feedback sandwich known as Ask Tell Ask by starting with asking the learner to 	self-assess followed by telling the learner your feedback, and ending with asking the learner for feedback 	regarding your teaching.
Example: Ask: “How do you feel you did with that procedure?” Wait for answer from learner;
	Tell: “I saw that you conducted an appropriate time out and followed the steps of the procedure in 	the appropriate order as we had discussed. You can work on explaining the procedure to the patient 	without using medical jargon.”
	Ask: “I would appreciate hearing from you how I can better teach you. What feedback do you have for 	me?”
Reinforcing Concept:
“Don’t practice until you get it right, practice until you can’t get it wrong.”
Language
Structure
Utilizing the “New” Feedback Sandwich
Culture

## Slide 18
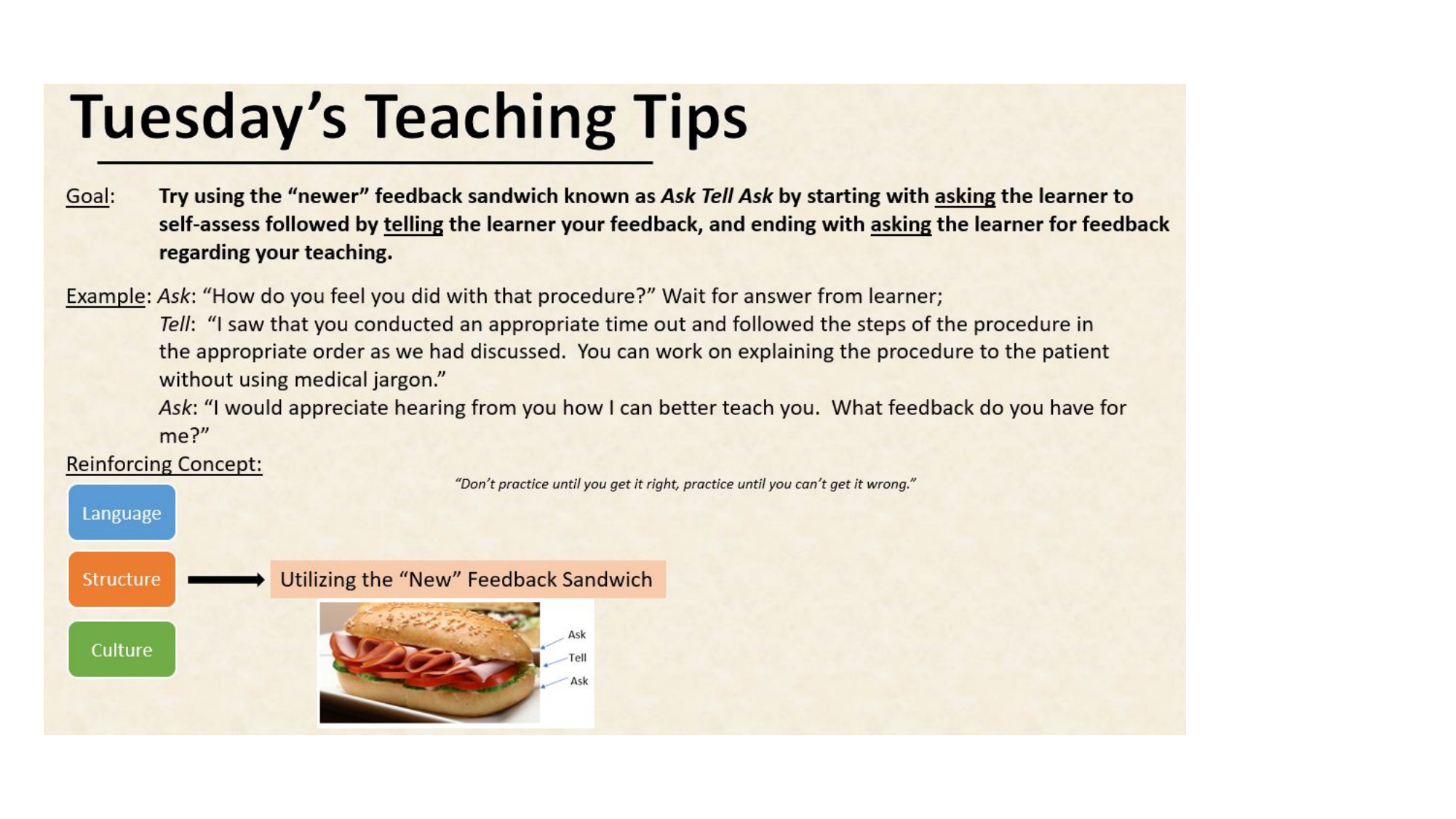

## Slide 19
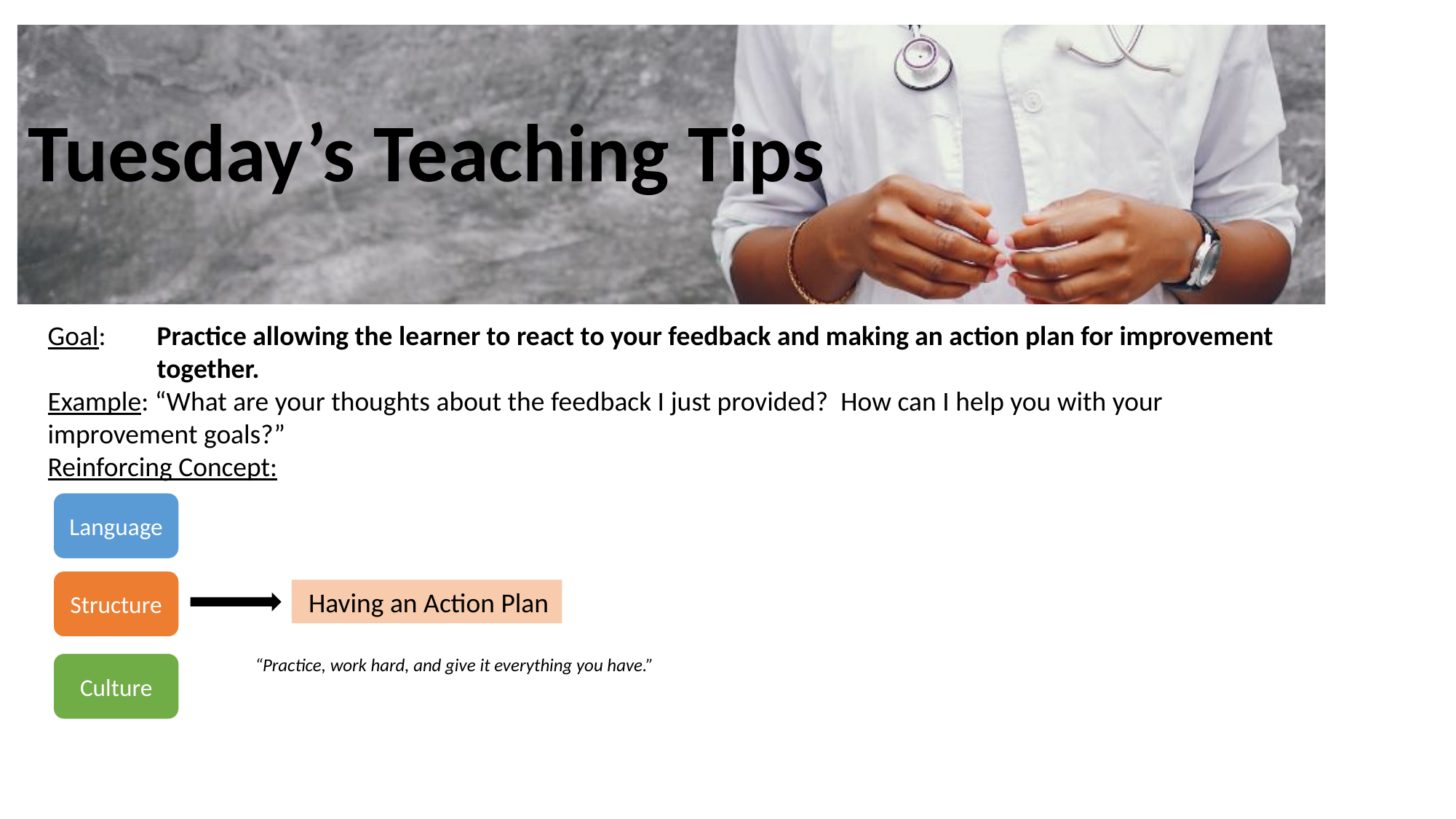

Tuesday’s Teaching Tips
Goal: 	Practice allowing the learner to react to your feedback and making an action plan for improvement 	together.
Example: “What are your thoughts about the feedback I just provided? How can I help you with your 	improvement goals?”
Reinforcing Concept:
Language
Structure
 Having an Action Plan
“Practice, work hard, and give it everything you have.”
Culture

## Slide 20
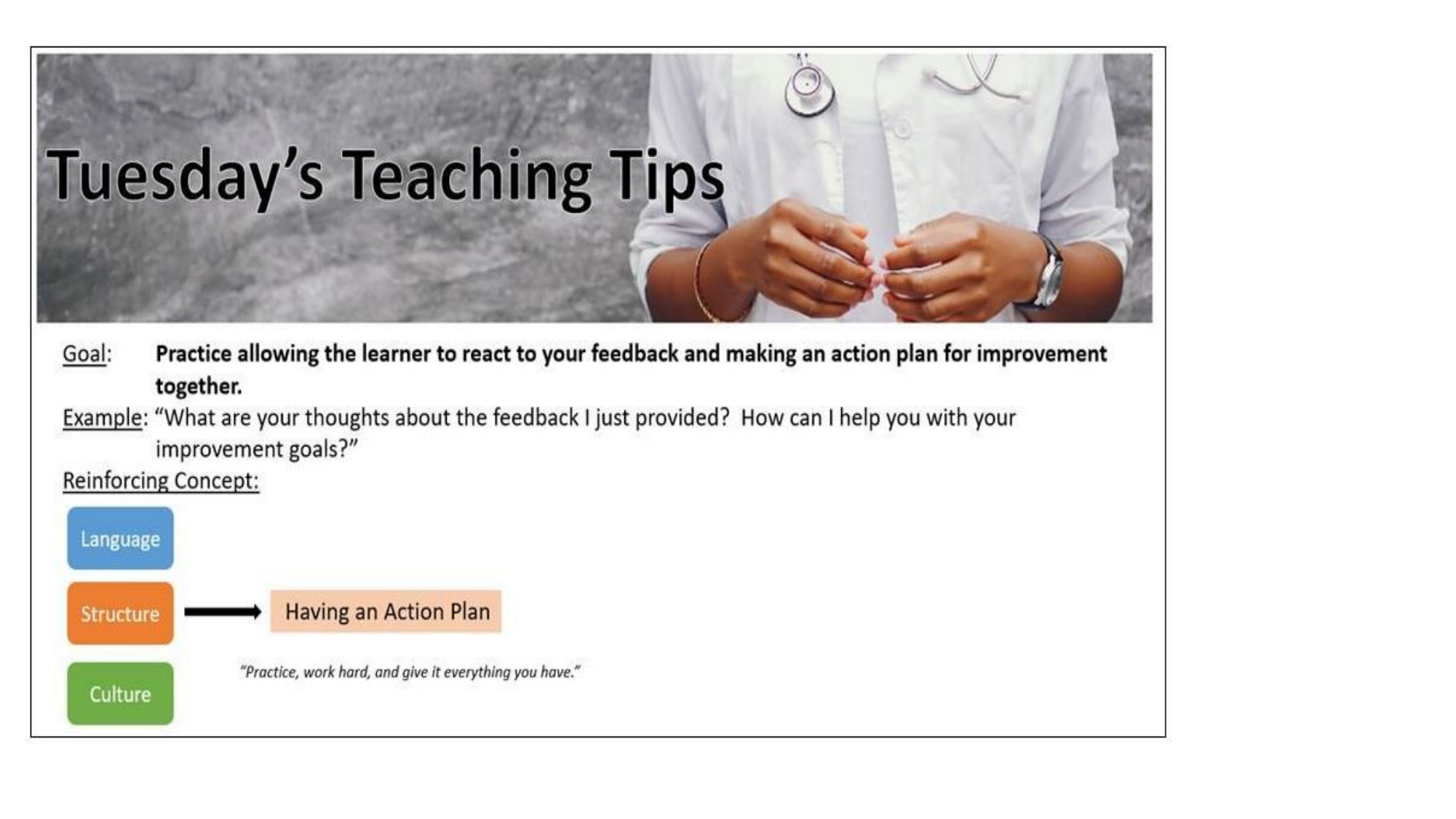

## Slide 21
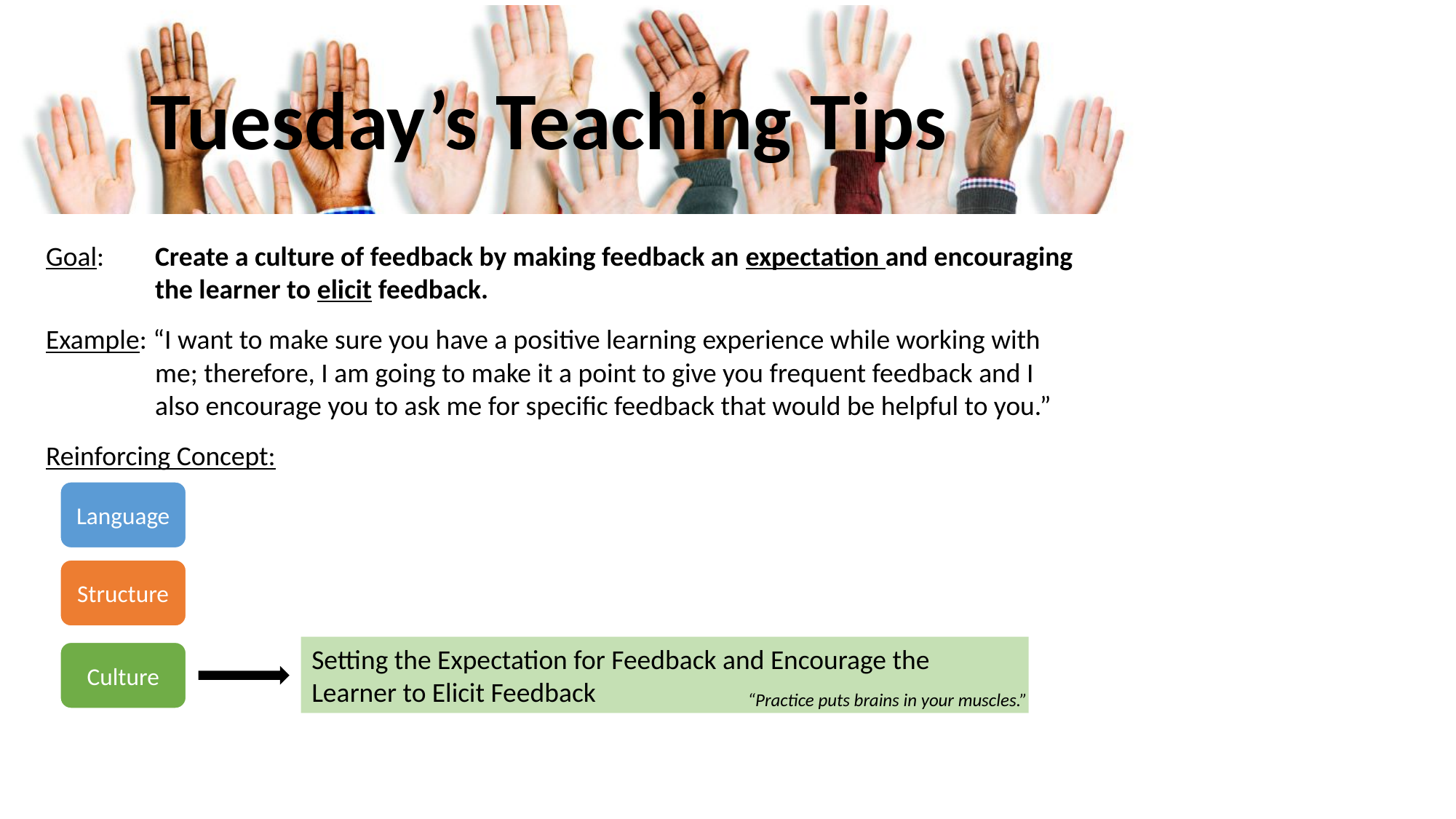

Tuesday’s Teaching Tips
Goal: 	Create a culture of feedback by making feedback an expectation and encouraging 	the learner to elicit feedback.
Example: “I want to make sure you have a positive learning experience while working with 	me; therefore, I am going to make it a point to give you frequent feedback and I 	also encourage you to ask me for specific feedback that would be helpful to you.”
Reinforcing Concept:
Language
Structure
Setting the Expectation for Feedback and Encourage the Learner to Elicit Feedback
Culture
“Practice puts brains in your muscles.”

## Slide 22
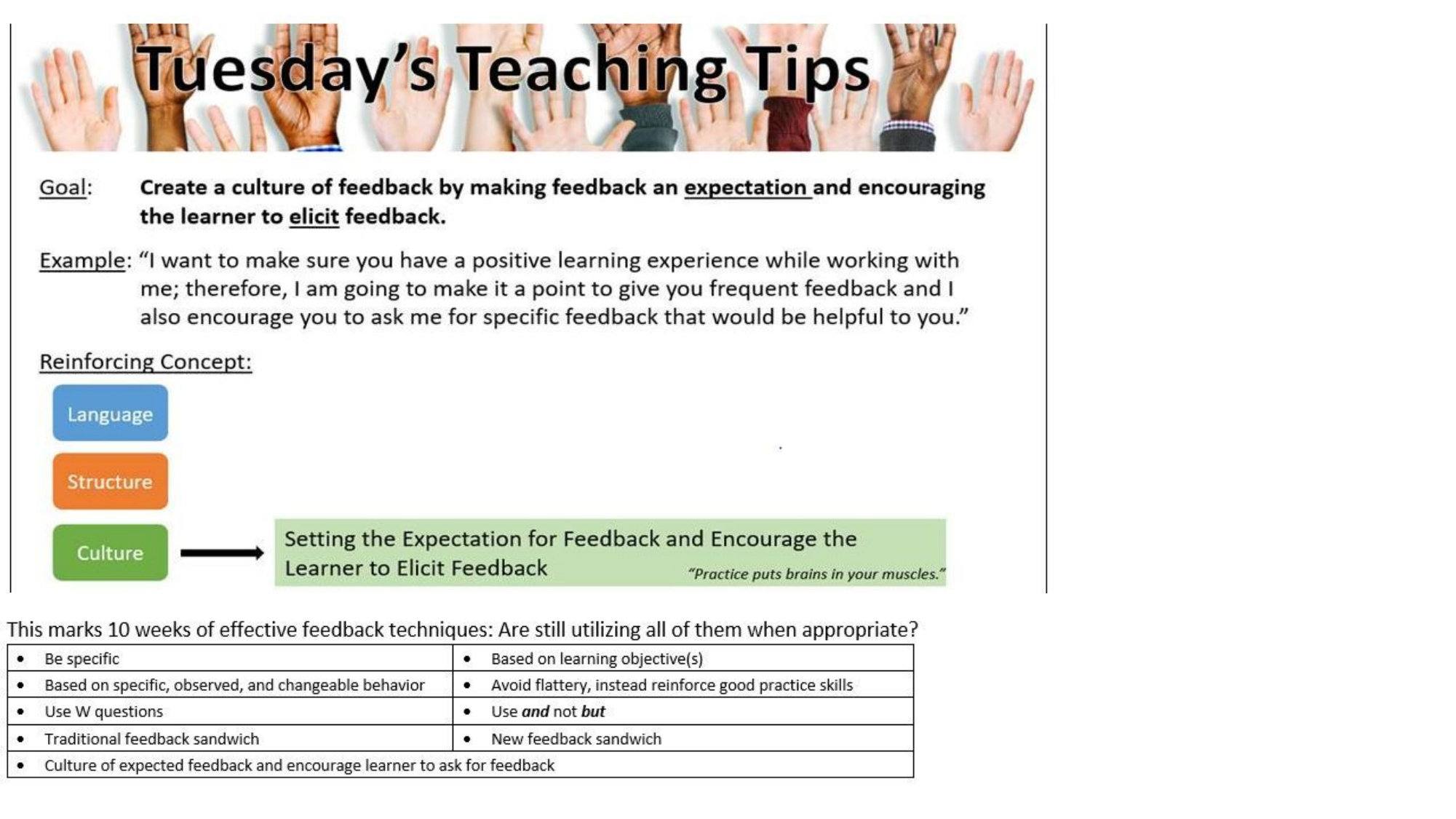

## Slide 23
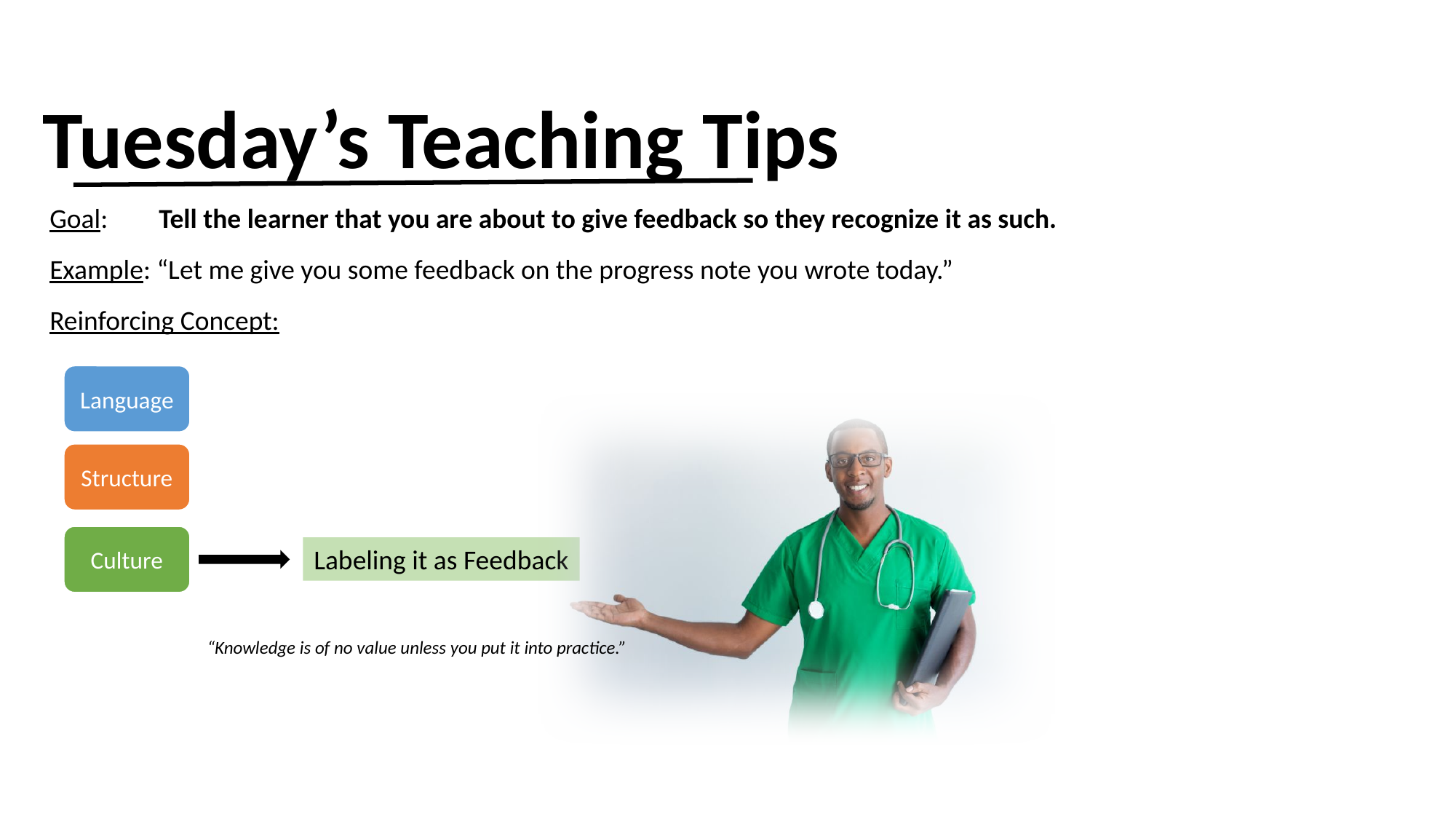

Tuesday’s Teaching Tips
Goal: 	Tell the learner that you are about to give feedback so they recognize it as such.
Example: “Let me give you some feedback on the progress note you wrote today.”
Reinforcing Concept:
Language
Structure
Culture
Labeling it as Feedback
“Knowledge is of no value unless you put it into practice.”

## Slide 24
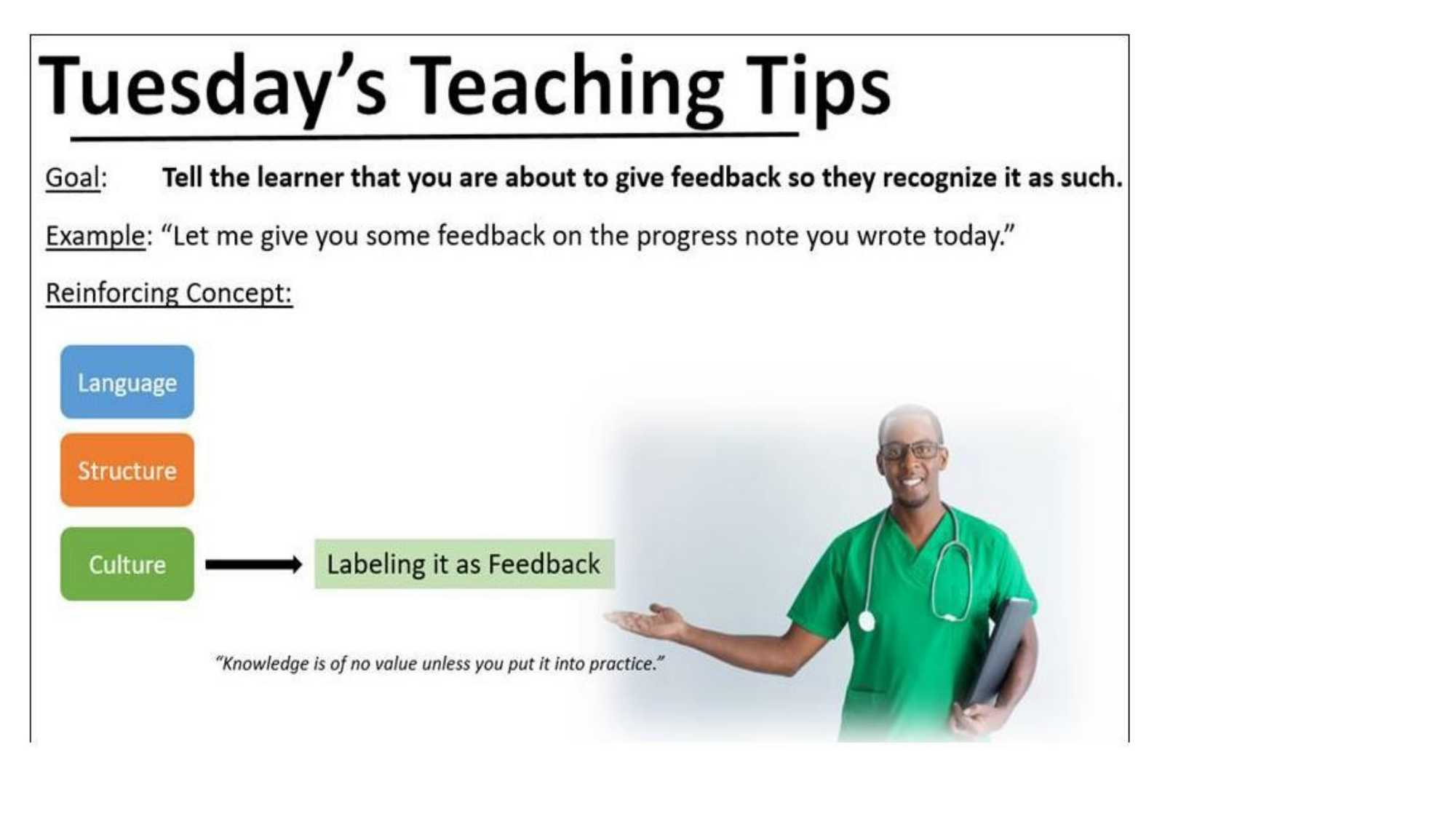

## Slide 25
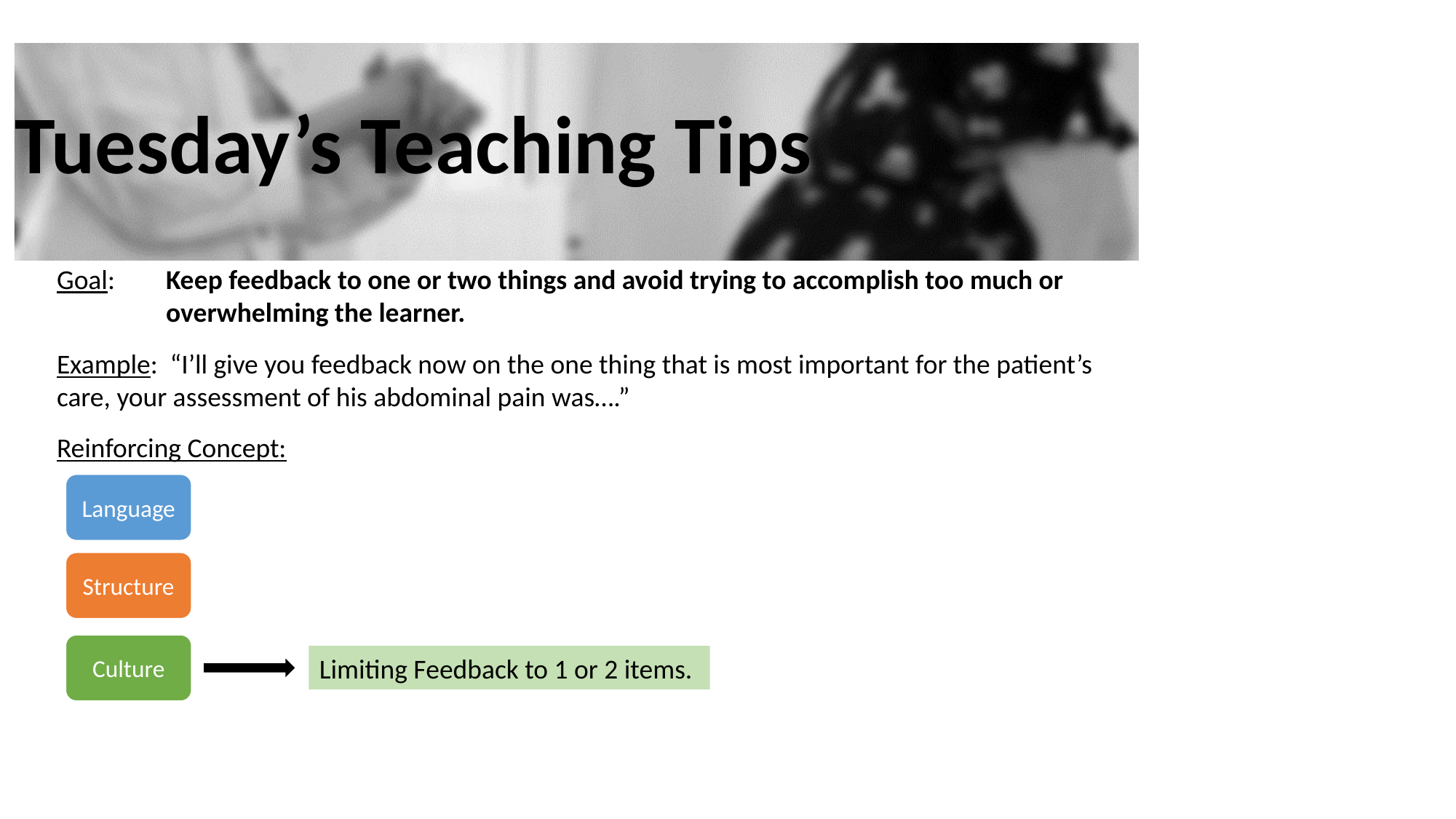

Tuesday’s Teaching Tips
Goal: 	Keep feedback to one or two things and avoid trying to accomplish too much or 	overwhelming the learner.
Example: “I’ll give you feedback now on the one thing that is most important for the patient’s care, your assessment of his abdominal pain was….”
Reinforcing Concept:
Language
Structure
Culture
Limiting Feedback to 1 or 2 items.

## Slide 26
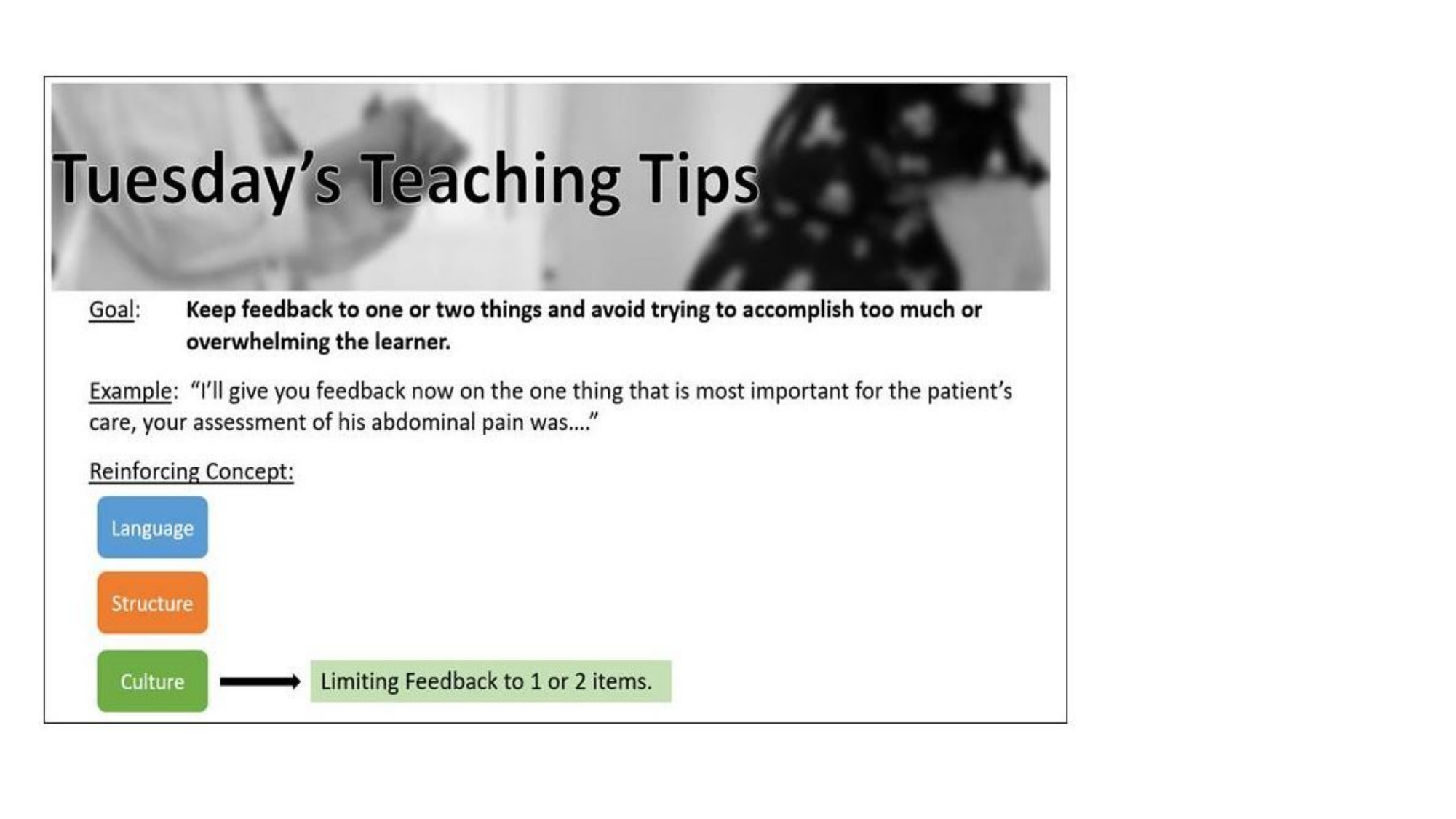

## Slide 27
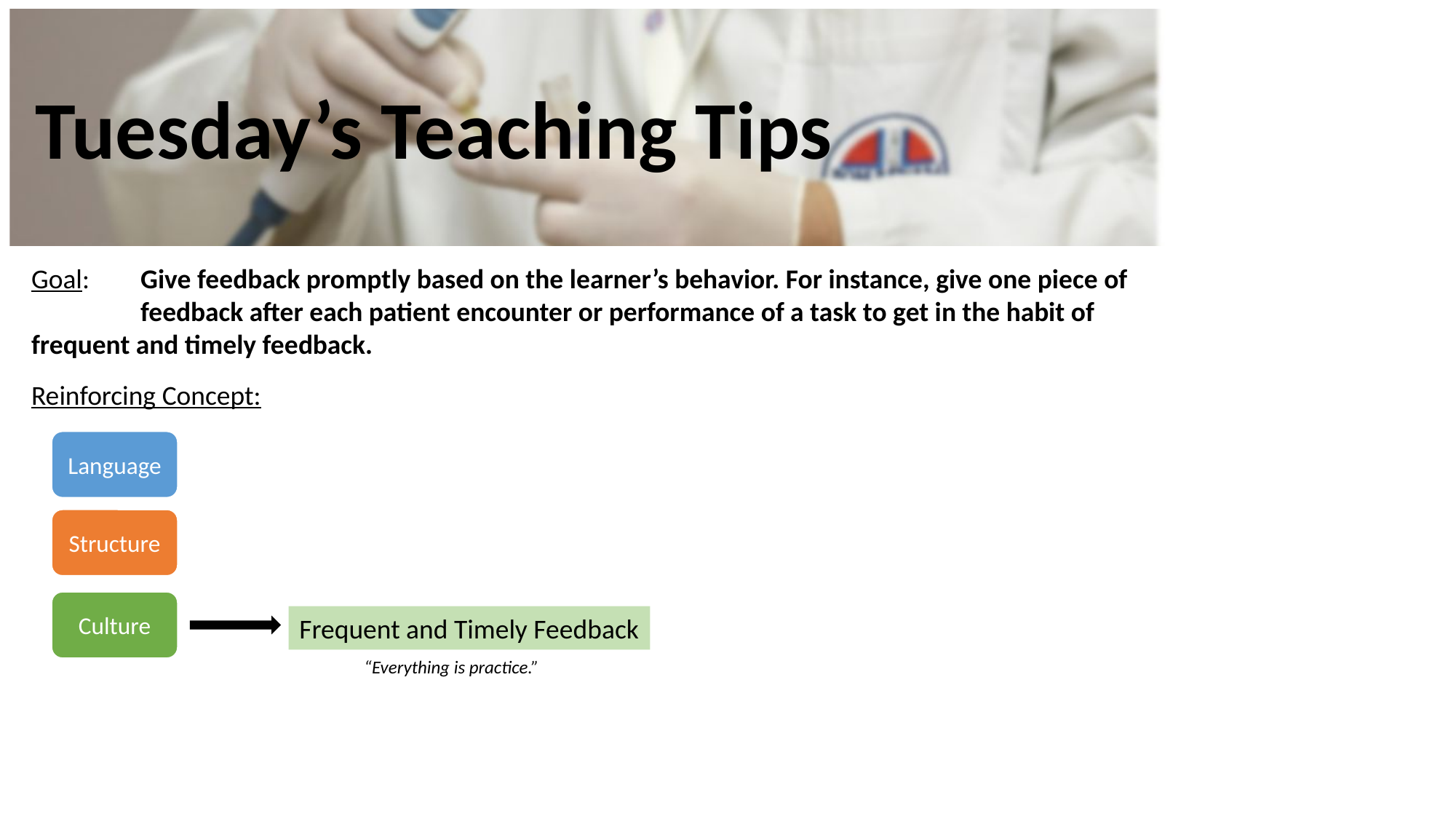

Tuesday’s Teaching Tips
Goal: 	Give feedback promptly based on the learner’s behavior. For instance, give one piece of 	feedback after each patient encounter or performance of a task to get in the habit of 	frequent and timely feedback.
Reinforcing Concept:
Language
Structure
Culture
Frequent and Timely Feedback
“Everything is practice.”

## Slide 28
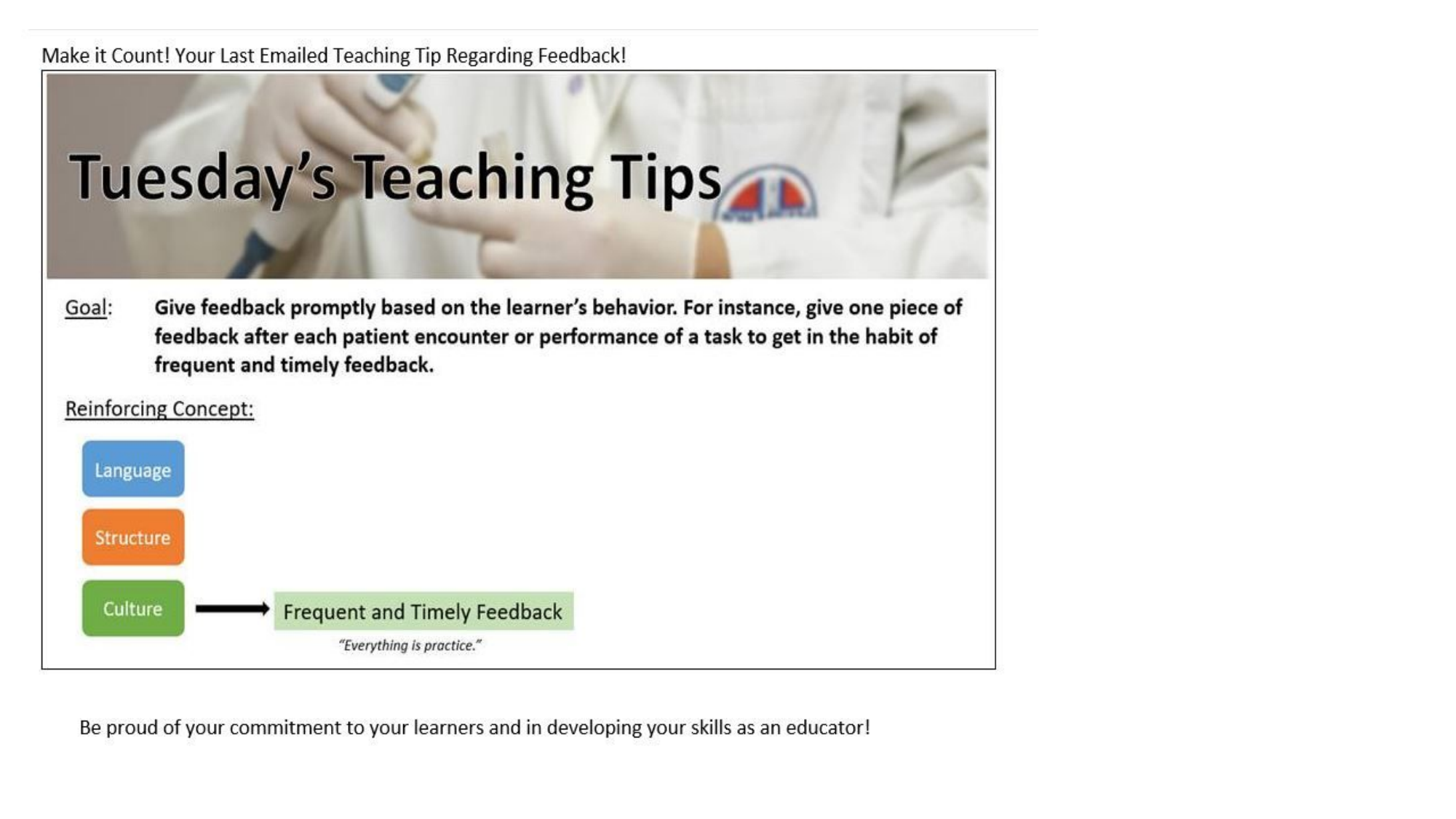

Supplement: Supplementary file 1 — Evaluation and Feedback Microlecture.m4vEmailed Tips.pptxProgram Announcement.pptxRegistration Form.docxProgram Directions.docxPreparatory Email.docxCertificate of Completion.docxPostmicrolecture Quiz.docxPostprogram Evaluation.docx [file mep_2374-8265.11281-s001.zip › B. Emailed Tips.pptx]
