# Supplementary material for: Tuesday's Teaching Tips—Evaluation and Feedback: A Spaced Education Strategy for Faculty Development
Source: MedEdPORTAL. 2022 Nov 22;18:11281. doi: 10.15766/mep_2374-8265.11281 (PMC9678823; doi:10.15766/mep_2374-8265.11281)
Supplement: Supplementary file 1 — Evaluation and Feedback Microlecture.m4vEmailed Tips.pptxProgram Announcement.pptxRegistration Form.docxProgram Directions.docxPreparatory Email.docxCertificate of Completion.docxPostmicrolecture Quiz.docxPostprogram Evaluation.docx [file mep_2374-8265.11281-s001.zip › C. Program Announcement.pptx]

## Slide 1
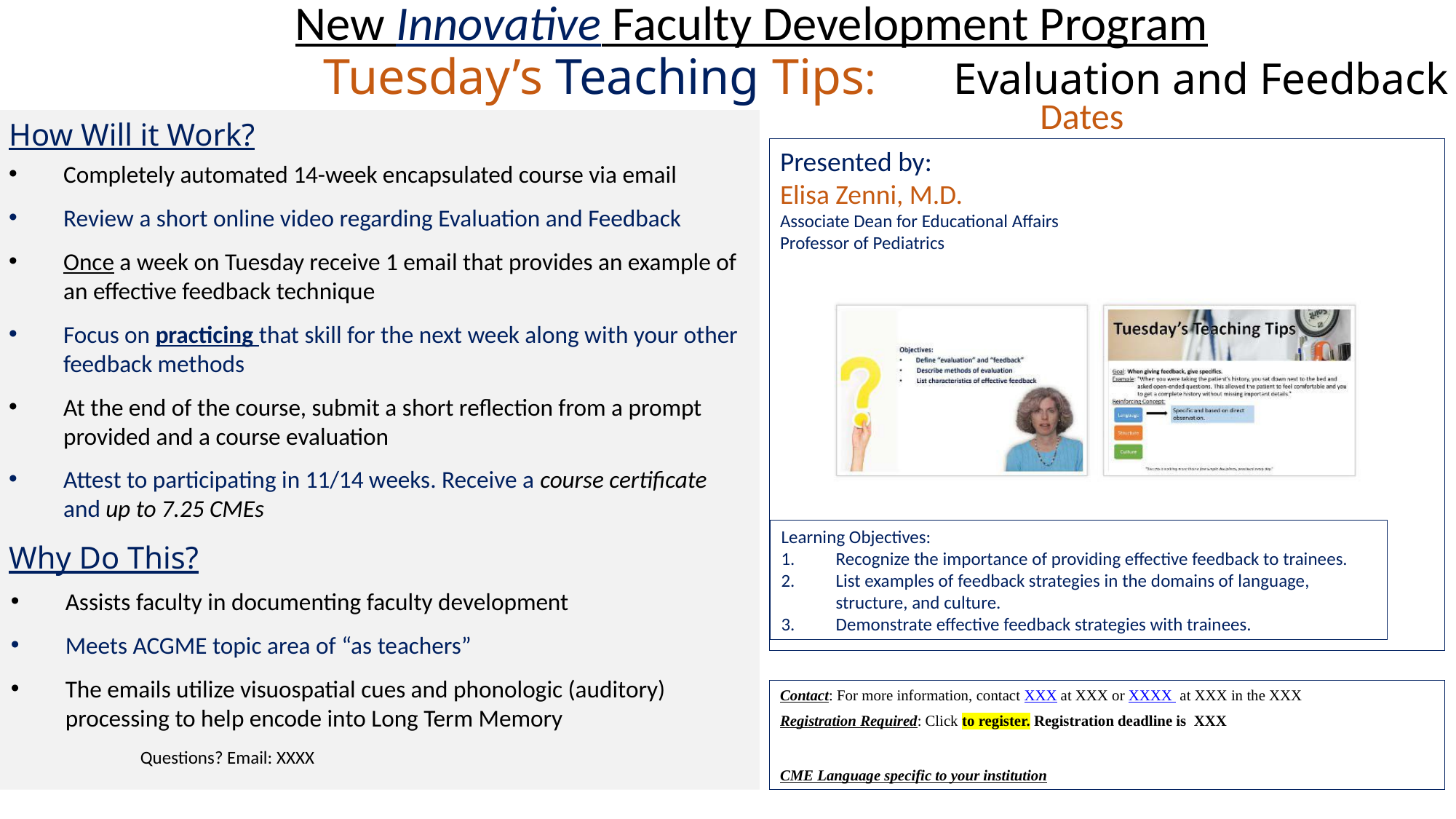

New Innovative Faculty Development Program
Tuesday’s Teaching Tips: Evaluation and Feedback
 Dates
How Will it Work?
Completely automated 14-week encapsulated course via email
Review a short online video regarding Evaluation and Feedback
Once a week on Tuesday receive 1 email that provides an example of an effective feedback technique
Focus on practicing that skill for the next week along with your other feedback methods
At the end of the course, submit a short reflection from a prompt provided and a course evaluation
Attest to participating in 11/14 weeks. Receive a course certificate and up to 7.25 CMEs
Presented by:
Elisa Zenni, M.D.
Associate Dean for Educational Affairs
Professor of Pediatrics
Learning Objectives:
Recognize the importance of providing effective feedback to trainees.
List examples of feedback strategies in the domains of language, structure, and culture.
Demonstrate effective feedback strategies with trainees.
Why Do This?
Assists faculty in documenting faculty development
Meets ACGME topic area of “as teachers”
The emails utilize visuospatial cues and phonologic (auditory) processing to help encode into Long Term Memory
 Questions? Email: XXXX
Contact: For more information, contact XXX at XXX or XXXX at XXX in the XXX
Registration Required: Click to register. Registration deadline is XXX
CME Language specific to your institution
