# Supplementary material for: Tuesday's Teaching Tips—Evaluation and Feedback: A Spaced Education Strategy for Faculty Development
Source: MedEdPORTAL. 2022 Nov 22;18:11281. doi: 10.15766/mep_2374-8265.11281 (PMC9678823; doi:10.15766/mep_2374-8265.11281)
Supplement: Supplementary file 1 — Evaluation and Feedback Microlecture.m4vEmailed Tips.pptxProgram Announcement.pptxRegistration Form.docxProgram Directions.docxPreparatory Email.docxCertificate of Completion.docxPostmicrolecture Quiz.docxPostprogram Evaluation.docx [file mep_2374-8265.11281-s001.zip › D. Registration Form.docx]

Tuesday’s Teaching Tips Registration

Thank you for your interest in Tuesday’s Teaching Tips. Completion of the form is required for your registration.

*Required

1. Please enter your LAST name.*

______________________________________

2. Please enter your FIRST name.*

______________________________________

3. Please enter your email.*

______________________________________

4. Please check your profession.*

*Check only one box.*

Faculty Physician

Affiliated Faculty Physician

Pharmacy (Contact your local program for CEU information)

Physical Therapy (Contact your local program for CEU information)

Nursing (This program does not have nursing CEUs)

Other (i.e. non-physician faculty, PA, etc.)

5. For physicians, please enter your department and/or training program.

___________________________________________________________________________

___________________________________________________________________________

___________________________________________________________________________

___________________________________________________________________________

___________________________________________________________________________

6. Please provide your institutional ID, if applicable.

______________________________________
