# Supplementary material for: Tuesday's Teaching Tips—Evaluation and Feedback: A Spaced Education Strategy for Faculty Development
Source: MedEdPORTAL. 2022 Nov 22;18:11281. doi: 10.15766/mep_2374-8265.11281 (PMC9678823; doi:10.15766/mep_2374-8265.11281)
Supplement: Supplementary file 1 — Evaluation and Feedback Microlecture.m4vEmailed Tips.pptxProgram Announcement.pptxRegistration Form.docxProgram Directions.docxPreparatory Email.docxCertificate of Completion.docxPostmicrolecture Quiz.docxPostprogram Evaluation.docx [file mep_2374-8265.11281-s001.zip › E. Program Directions.docx]

**Program Directions: Welcome to Tuesday’s Teaching Tips Program- Evaluation and Feedback**

Directions:

**Background:**

We implemented Tuesday’s Teaching Tips (TTT) as a Continuing Medical Education (CME) 14-week faculty development program. TTT utilizes spaced education, as it first provides a foundational micro-lecture on evaluation and feedback, then is followed by weekly emailed statements that are phrased as skills-based tips to implement in the clinical learning environment by faculty with trainees. It is expected that the weekly tip provided is “practiced” by faculty for the week, and then the next tip, etc. The tips also utilizes visuospatial cues (connecting back to the micro-lecture for reinforcement) and because faculty practice the tips, phonologic. Both aspects assist with long-term memory encoding.

Step 1. Apply for CME credit.

a. Micro-lecture. To use our micro-lecture, as the tips are specifically designed with visuospatial cues in that lecture, utilize the MP4 (Appendix A). If you wish to have CMEs, you will need to apply for CMEs through your own sources. Dr. Zenni, who provided the micro-lecture, is willing to sign a disclosure form as the presenter (elisa.zenni@jax.ufl.edu). The micro-lecture is .25 CME credit (it is about 15 minutes) and each weekly tip and practice is .5 CME credit (30 minutes) for a total of 7.25 CME credits for complete participation. After watching the micro-lecture, faculty take the quiz (Appendix H). At the end of the program, the post-program evaluation is sent where faculty attest to practicing 80% of the tips, complete the CME evaluation with an added reflective statement (Appendix I).

Information we submitted for CME.


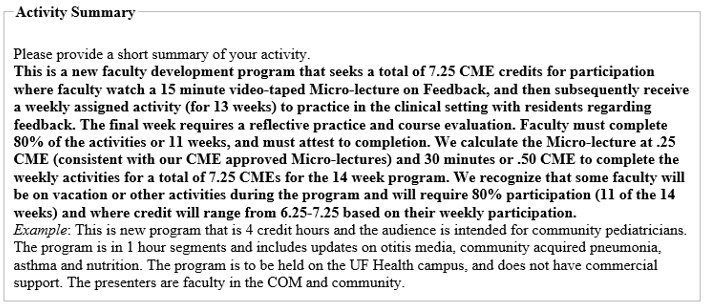


Step 2. Ensure the set-up in your email system.

a. After faculty sign-up to participate, create a list-serve with everyone’s email. On Monday afternoons, set-up the email for the next day to be sent automatically. Be cautious with trying to set-up all of the TTTs in advance to have them sent out on a schedule. If your email system does an IT update or reboot, the “automatic” email may not happen. It is safest to set up each email the day before it needs to be sent. You can add yourself to the email list serve to ensure that the emails are delivered at 7:00 a.m. each Tuesday.

b. Directions for the “read receipt.” The “read receipt” serves as a proxy for attendance. Set-up your email system so that you automatically receive the receipt once faculty open the email. Include a back-up approach allowing faculty to email you to document that they read the email, if the read receipt does not work. You can track attendance in an Excel Spreadsheet.

c. The email tips:

Week One: assignment was to watch the micro-lecture, take the quiz and to practice the first tip. (Appendix A; Appendix H). The Power Point has the original email mock-ups (Appendix B). The Power Point has clearer resolution. We used the “snipping tool” to transfer the photo to the email. This decreased some of the resolution, which is why they are a little blurry. *Below is a snap shot of each week’s tip.

Step 3. Sign-up for participants.

a. Once CME is approved, send out an email with a flyer and announce the program at relevant institutional meetings (Appendix C). Faculty can sign-up electronically through a link to a Google form (Appendix D). You may also consider creating an internal web-page for the program with the registration link.

Step 4. Send preparatory email with directions. The Friday before the program starts, send a preparatory email with overall program directions to help set expectations (Appendix F).

Emailed statements on next page:

***Week One:**


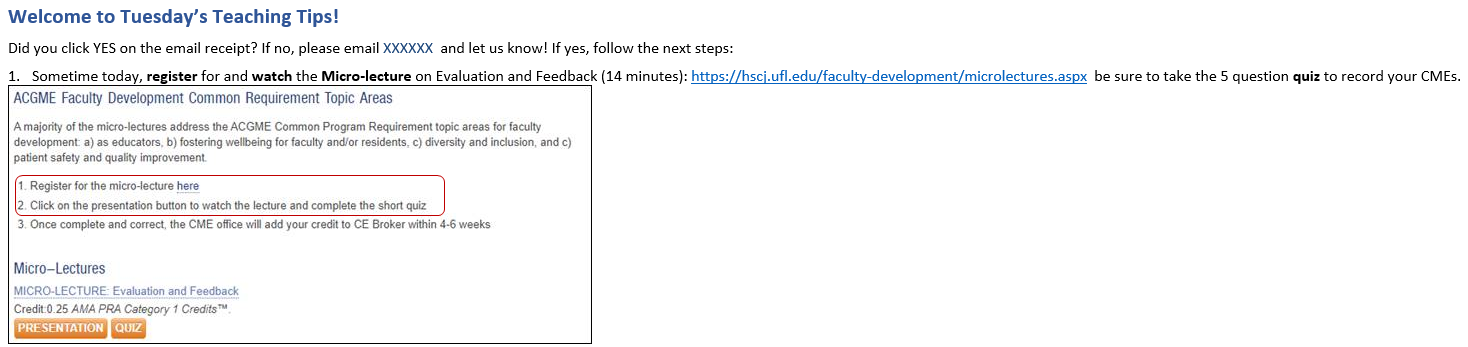


Provide link to the micro-lecture


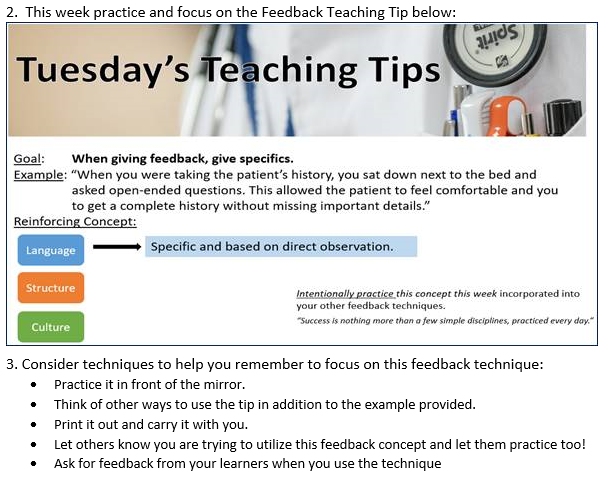


*Weeks 2-13 as Thumb-nails for easy reference to the PPT.

| Week 2 **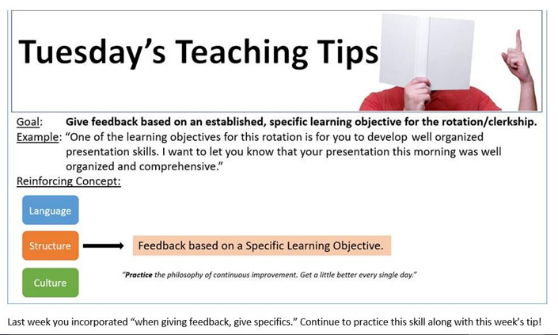** | Week 3  **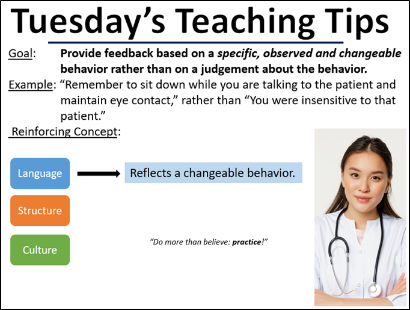**  **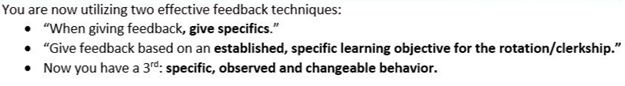** | 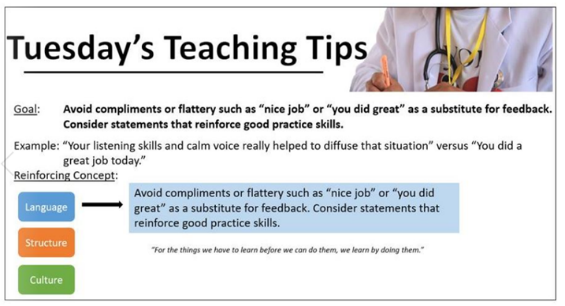Week 4  How are you doing incorporating previous weeks’ tips? What are they? Do you remember how they were introduced in the video? |
| --- | --- | --- |
| Week 5  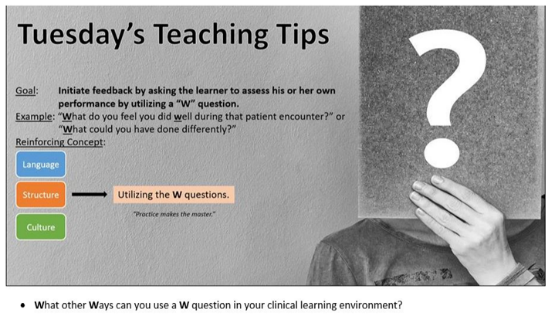 | Week 6  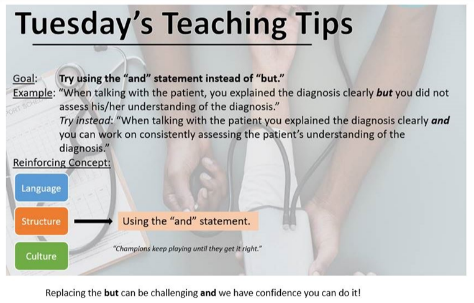 | 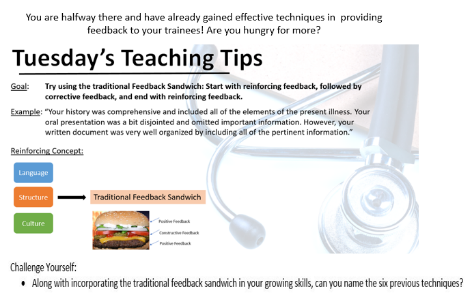Week 7 |
| 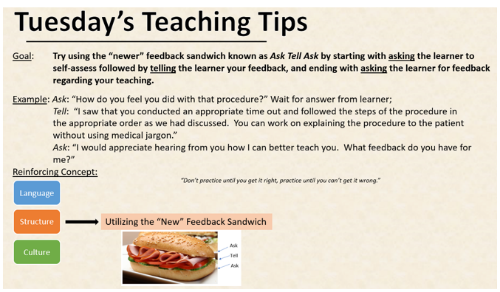Week 8 | Week 9  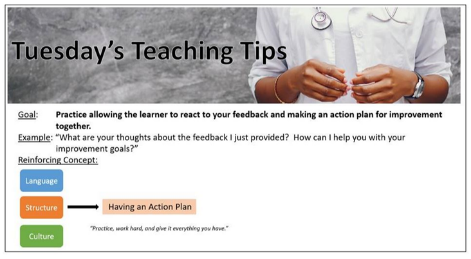 | Week 10  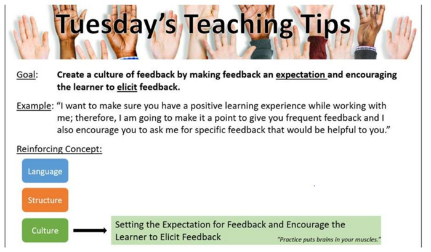  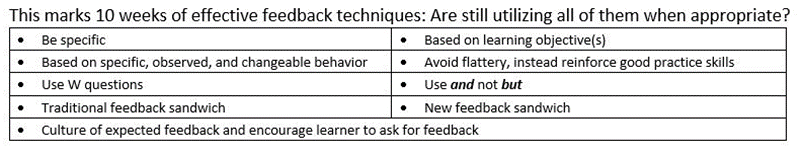 |
| Week 11  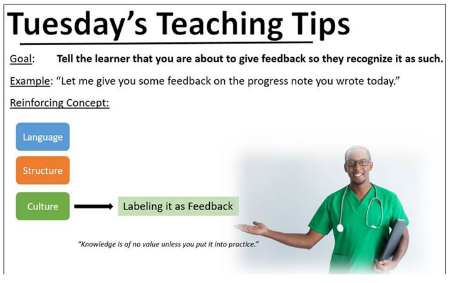 | Week 12  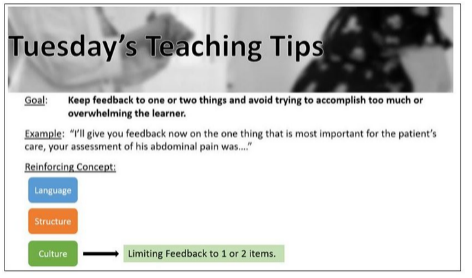 | 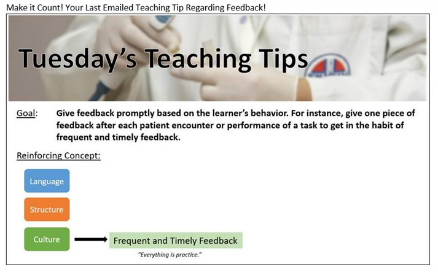Week 13 |

Step 5. Create the evaluation (both the CME required evaluation and the program reflection statement) in a Google Form that participants can submit electronically (Appendix I).

Step 6. Provide a certificate (Appendix G) to participants who attested to participating in at least 11 out of the 13 weeks and completed the program evaluation with self-reflection.

**Final Item to Consider:**

**Practice!** It is helpful to do several dry runs prior to the start, ensuring that the email system and read receipts work and problem solve in advance.
