# Supplementary material for: Tuesday's Teaching Tips—Evaluation and Feedback: A Spaced Education Strategy for Faculty Development
Source: MedEdPORTAL. 2022 Nov 22;18:11281. doi: 10.15766/mep_2374-8265.11281 (PMC9678823; doi:10.15766/mep_2374-8265.11281)
Supplement: Supplementary file 1 — Evaluation and Feedback Microlecture.m4vEmailed Tips.pptxProgram Announcement.pptxRegistration Form.docxProgram Directions.docxPreparatory Email.docxCertificate of Completion.docxPostmicrolecture Quiz.docxPostprogram Evaluation.docx [file mep_2374-8265.11281-s001.zip › F. Preparatory Email.docx]

**Welcome to Tuesday’s Teaching Tips**

Congratulations for signing-up for this innovative program! To make sure everyone is ready, here are some directions:

**How the program works:**

- Program runs for 14 weeks: (Dates)
- Week One
  - You’ll receive the link **to register** for the online video regarding Evaluation and Feedback, **watch it first,** **and then take the 5-question quiz.** This is important for CME credit and credit for the program for tracking purposes.
  - You will also receive in this email, the first teaching tip to start practicing in the clinical setting.
    - Practicing the feedback concepts is a very important part of program.
    - The emails are designed with embedded visual cues from the video and practicing is an auditory reinforcement; together, these techniques will help to implant these concepts into long term memory for use.
- The emails you receive will come from a XXXXX email and will be labelled as Tuesday’s Teaching Tips
- **You must click “YES” for the “Read ME” receipt.** This will notify our office that you have opened the email. If you do not accept the email upon opening it, it will not come back up again, and you will have to email XXXXXXX to let us know ***that week*** that you read the email. This is how we will track attendance.
- Please do not reply ALL to the emails sent, it will go to everyone on the list serve. Email us directly with questions or comments.

**Weeks 2-13:**

- You will receive one email a week on Tuesday’s with a new teaching tip.
- Again, click “yes” on the receipt email.
- Intentionally practice the teaching tip.

**Week 14:** As part of CME and Program Certificate requirements you’ll receive:

- A link that asks for you to complete the program evaluation (CME questions), an attestation statement that you participated in 11 of the 14 weeks (80%), and for your reflection regarding lessons learned from the program.

Other Ideas to consider to help you remember to practice the teaching tip:

- Practice it in front of the mirror.
- Think of other ways to use the tip in addition to the example provided.
- Print it out and carry it with you.
- Let others know you are trying to utilize this feedback concept and let them practice too!
