# Supplementary material for: Tuesday's Teaching Tips—Evaluation and Feedback: A Spaced Education Strategy for Faculty Development
Source: MedEdPORTAL. 2022 Nov 22;18:11281. doi: 10.15766/mep_2374-8265.11281 (PMC9678823; doi:10.15766/mep_2374-8265.11281)
Supplement: Supplementary file 1 — Evaluation and Feedback Microlecture.m4vEmailed Tips.pptxProgram Announcement.pptxRegistration Form.docxProgram Directions.docxPreparatory Email.docxCertificate of Completion.docxPostmicrolecture Quiz.docxPostprogram Evaluation.docx [file mep_2374-8265.11281-s001.zip › G. Certificate of Completion.docx]

Your LOGO

Certificate of Achievement

presented to

**Doctor Name, M.D.**

for completion of the

Tuesday’s Teaching Tips

Evaluation and Feedback

DATE:

*Senior Associate Dean for Educational Affairs Dean, College of Medicine –*
