# Supplementary material for: Tuesday's Teaching Tips—Evaluation and Feedback: A Spaced Education Strategy for Faculty Development
Source: MedEdPORTAL. 2022 Nov 22;18:11281. doi: 10.15766/mep_2374-8265.11281 (PMC9678823; doi:10.15766/mep_2374-8265.11281)
Supplement: Supplementary file 1 — Evaluation and Feedback Microlecture.m4vEmailed Tips.pptxProgram Announcement.pptxRegistration Form.docxProgram Directions.docxPreparatory Email.docxCertificate of Completion.docxPostmicrolecture Quiz.docxPostprogram Evaluation.docx [file mep_2374-8265.11281-s001.zip › H. Postmicrolecture Quiz.docx]

Evaluation and Feedback Micro-Lecture Quiz

1. **Evaluation is defined as giving learners information about their current performance. T F** Answer: False. Evaluation is defined as the process by which the teacher assesses the learners’ knowledge, skills and attitudes, based on criteria related to educational goals.
2. **Feedback is defined as the process by which teachers assess the learners’ knowledge, skills, and attitudes.” T F**

Answer: False. Feedback is defined as the process of giving learners information about their current performance so that they may improve it in the future.

1. **The corner stone of evaluation is direct observation. T F**

Answer: True. Direct observation is the primary method of evaluating learners.

1. **Feedback should reflect a changeable behavior. T F**

Answer: True. Feedback should be specific, based on direct observation, non-judgmental, and reflect a changeable behavior.

1. **A way to structure your feedback is to utilize the “and” statement versus the “but” statement to decrease defensiveness in your learner. T F**

Answer: True. Using “and” instead of “but” when giving feedback implies that everyone does things well and also can improve.
