# Supplementary material for: Tuesday's Teaching Tips—Evaluation and Feedback: A Spaced Education Strategy for Faculty Development
Source: MedEdPORTAL. 2022 Nov 22;18:11281. doi: 10.15766/mep_2374-8265.11281 (PMC9678823; doi:10.15766/mep_2374-8265.11281)
Supplement: Supplementary file 1 — Evaluation and Feedback Microlecture.m4vEmailed Tips.pptxProgram Announcement.pptxRegistration Form.docxProgram Directions.docxPreparatory Email.docxCertificate of Completion.docxPostmicrolecture Quiz.docxPostprogram Evaluation.docx [file mep_2374-8265.11281-s001.zip › I. Postprogram Evaluation.docx]

Tuesday’s Teaching Tips: Evaluation and Feedback

As part of meeting continuing education credit requirements, please complete the following evaluation. Your comments are very important to us! All answers will be de-identified from course directors by administrative staff.

*Required

1. First name, last name, and license number:*

______________________________________

2. Will information gained from this program result in enhancing optimal patient care and/or medical education? *

*Check only one box*.

Yes

No

3. If yes, please list change(s) you intend to make in your practice or teaching as a result of this program.

___________________________________________________________________________

___________________________________________________________________________

___________________________________________________________________________

___________________________________________________________________________

___________________________________________________________________________

4. Please rate your confidence in implementing these changes: *

*Check only one box*

High Confidence

Moderate Confidence

Low/No Confidence

N/A

5. Please identify any of the following barriers you perceive in implementing these changes (select all that apply):*

*Check all that apply.*

Cost

Lack of time

Insurance/Reimbursement issues

Patient or trainee compliance issues

Lack of consensus of professional guidelines

N/A

6. How will you address these barriers to implement changes in knowledge and behavior?

___________________________________________________________________________

___________________________________________________________________________

___________________________________________________________________________

___________________________________________________________________________

___________________________________________________________________________

7. The material was presented at an appropriate level: *

*Check only one box*

Strongly Agree

Agree

Neutral

Disagree

Strongly Disagree

8. I have gained knowledge that will improve patient care (through teaching/training): *

*Check only one box*

Strongly Agree

Agree

Neutral

Disagree

Strongly Disagree

9. The program met my expectations in accomplishing the stated educational objectives: *

*Check only one box*

Strongly Agree

Agree

Neutral

Disagree

Strongly Disagree

10. The program content was objective, balanced, and free from commercial bias or influence: *

*Check only one box*

Strongly Agree

Agree

Neutral

Disagree

Strongly Disagree

11.Your overall rating of the quality of the education offered at this program: *

*Check only one box*

Excellent

Good

Average

Fair

Poor

12. Did the coronavirus affect your ability to participate in Tuesday Teaching Tips? *

*Check only one box*

Yes

No

Somewhat

13. If yes, please provide a brief explanation, as we may be able to make adjustments in the future:

___________________________________________________________________________

___________________________________________________________________________

___________________________________________________________________________

___________________________________________________________________________

___________________________________________________________________________

14. I would like to see other Tuesday Teaching Tips topics offered such as creating a positive learning climate, active learning strategies, and teaching skills techniques.

*Check only one box*

Yes

No

15. Other suggested topics for Medical Education:

___________________________________________________________________________

___________________________________________________________________________

___________________________________________________________________________

__________________________________________________________________________

16. Additional comments to help improve the program:

___________________________________________________________________________

___________________________________________________________________________

___________________________________________________________________________

___________________________________________________________________________

___________________________________________________________________________

In order to earn the certificate of completion the following reflective statement must be answered and the attestation checked.

Tuesday’s Teaching Tips Reflective Statement:

17. Please describe how this program has improved your overall knowledge and skills related to evaluation and feedback when working with your trainees. In order to meet CME requirements, please provide specific examples. Your reflection should be between 250-500 words.

___________________________________________________________________________

___________________________________________________________________________

___________________________________________________________________________

___________________________________________________________________________

___________________________________________________________________________

18. “I attest to completing 11 of the 13 weeks of the Tuesday’s Teaching Tips to earn the Course Certificate.”

*Check all that apply.*

Yes

19. I was unable to complete 11 of the 13 weeks, please calculate CMEs based on my attendance/read receipts.

*Check all that apply.*

Yes
