# Supplementary material for: Tuesday's Teaching Tips—Evaluation and Feedback: A Spaced Education Strategy for Faculty Development
Source: MedEdPORTAL. 2022 Nov 22;18:11281. doi: 10.15766/mep_2374-8265.11281 (PMC9678823; doi:10.15766/mep_2374-8265.11281)
Supplement: Supplementary file 1 — Evaluation and Feedback Microlecture.m4vEmailed Tips.pptxProgram Announcement.pptxRegistration Form.docxProgram Directions.docxPreparatory Email.docxCertificate of Completion.docxPostmicrolecture Quiz.docxPostprogram Evaluation.docx [file mep_2374-8265.11281-s001.zip › Educational_Summary_Report_11281.pdf]

MedEdPORTAL® The AAMC Journal of Teaching and Learning Resources

Original Publication

Open Access

Tuesday's Teaching Tips—Evaluation and Feedback: A Spaced Education Strategy for Faculty Development

Colleen Kalynych, EdD*, Linda Edwards, MD, Denise West, MA, Charity Snodgrass, Elisa Zenni, MD

*Corresponding author: colleen.kalynych@jax.ufl.edu

Abstract

Introduction: The AGGME requires faculty to participate annually in faculty development sessions. Barriers to this requirement include faculty having a lack of time and not perceiving benefits to participating. Effective evaluation and feedback are integral to resident training. Faculty often feel ill prepared to deliver feedback, and residents find accepting and recognizing feedback challenging. We provided faculty with a spaced education program via email that used cognitive theory of multimedia learning solutions in instructional design. **Methods:** The 14-week program consisted of one microlecture and 13 skills-based teaching tips. One tip reinforcing knowledge and skills from the microlecture was emailed each week for faculty to practice in the clinical environment with trainees. Participants completed a short quiz, course evaluation, and self-reflection. The new world Kirkpatrick model was used for program evaluation. **Results:** Fifty-two physician participants received credit for participating; 34 completed the entire course. Of the 34, 32 (94%) identified at least one effective feedback technique, and 27 (79%) were able to define evaluation and recognize observation as the cornerstone of evaluation. Out of the 15 effective feedback characteristics taught, 13 (87%) were identified. Fifty-one participants (98%) rated the program as good/excellent, 52 (100%) wanted more Tuesday's Teaching Tips programs, and the majority recognized change in knowledge and/or skills. **Discussion:** Participants rated the spaced education program as good/excellent and were able to meet the course objectives. This teaching strategy for faculty development was well received, as it was easily accessible and implemented in the clinical learning environment with trainees.

Keywords:
Evaluation, Spaced Education, Clinical Teaching/Bedside Teaching, Faculty Development, Feedback

Educational Objectives

By the end of this activity, learners will be able to:

1. Define evaluation and feedback.
2. Describe methods of evaluation.
3. List characteristics of effective feedback.
4. Assess change in knowledge and skills related to evaluation and feedback.

Introduction

The ACGME requires faculty development programs to improve the knowledge, skills, and behaviors of faculty as educators.[1] However, barriers to faculty participating in faculty development are well documented and include a lack of time due to clinical responsibilities, a lack of protected time, competing continuing education needs within their own specialty, a perceived lack of recognition and financial reward for teaching, inconvenient faculty development sessions, a tendency for faculty to underestimate the need for and potential benefits of training, underestimation of the usefulness of teaching skills compared to clinical skills, and concern that teacher training is unrelated to teaching excellence.[2-6] Paramount to resident training is effective evaluation (assessment) of the resident's knowledge and performance as well as timely feedback to reinforce and/or correct medical knowledge and/or behavior. Effective evaluation and feedback enable resident growth in terms of formative assessment and assist with achievement of clinical competence (summative assessment).[7-9] However, both faculty and residents report widespread dissatisfaction regarding faculty skills in effective evaluation and feedback and resident recognition and acceptance of each when presented.[10-15] Additionally, faculty continue to report discomfort in giving feedback and feel ill prepared to accomplish this task.[16-22]

Citation:
Kalynych C, Edwards L, West D, Snodgrass C, Zenni E. Tuesday's Teaching Tips—Evaluation and Feedback: a spaced education strategy for faculty development. *MedEdPORTAL*. 2022;18:11281.
https://doi.org/10.15766/mep_2374-8265.11281

Copyright © 2022 Kalynych et al. This is an open-access publication distributed under the terms of the Creative Commons Attribution license.

MedEdPORTAL® The AAMC Journal of Teaching and Learning Resources

In an effort to address faculty development regarding evaluation and feedback and to take into account faculty time constraints, we developed an innovative faculty development program entitled Tuesday's Teaching Tips—Evaluation and Feedback (TTT) that built upon programs by Matzie and colleagues[23] and Pernar and colleagues.[24] The Matzie study provided spaced education to surgical residents as part of a residents-as-teachers program to improve feedback to medical students, and the Pernar study sought to improve surgical faculty's teaching skills with medical students. In both studies, the target population received weekly emails in two cycles over 9 months with a brief statement and, in some instances, an example for implementation. Spaced education is a type of distributive practice by which information or procedures are presented and repeated in subsequent reviews to enhance long-term retention of knowledge and transfer of skills.[25] A *MedEdPORTAL* search using the term *evaluation and feedback* yielded over 1,000 publications, most of which described workshops or online modules, but none that appeared to involve an email approach to faculty development. Similarly, a *MedEdPORTAL* search for the term *spaced education* did not reveal relevant publications utilizing a spaced education approach to faculty development.

A key difference from the Matzie and Pernar programs is that ours is based on multimedia learning, in which learning occurs from words and pictures versus words only. We sought not only to provide spaced education in the form of weekly email tips regarding evaluation and feedback (words) but also to incorporate a microlecture (video, pictures, and words) establishing foundational knowledge upon which to base the spaced education emails and, further, to utilize cognitive theory of multimedia learning (CTML) solutions to address cognitive load concerns.[26] Mayer and others have suggested the importance of taking into account cognitive load to address the finite quantity of cognitive processing the mind can accomplish in both the verbal and visual channels in working memory.[27-29] Yet there is a balance in being able to hold the learner's attention and draw connections back to previous knowledge and experiences or information just learned (e.g., spaced education, learning and working memory tenets). The use of visual cues is a CTML solution that utilizes coloring, arrows, borders, highlighting, bolding, and other visual cues to capture or guide the learner's attention and has been shown to be effective in learning.[26] For example, whereas the Matzie and Pernar studies' emails consisted of text (words) only (top left panel of the Figure), our design (top right and bottom panels of the Figure) shows the CTML solution technique of using visual cues or signaling. This technique is

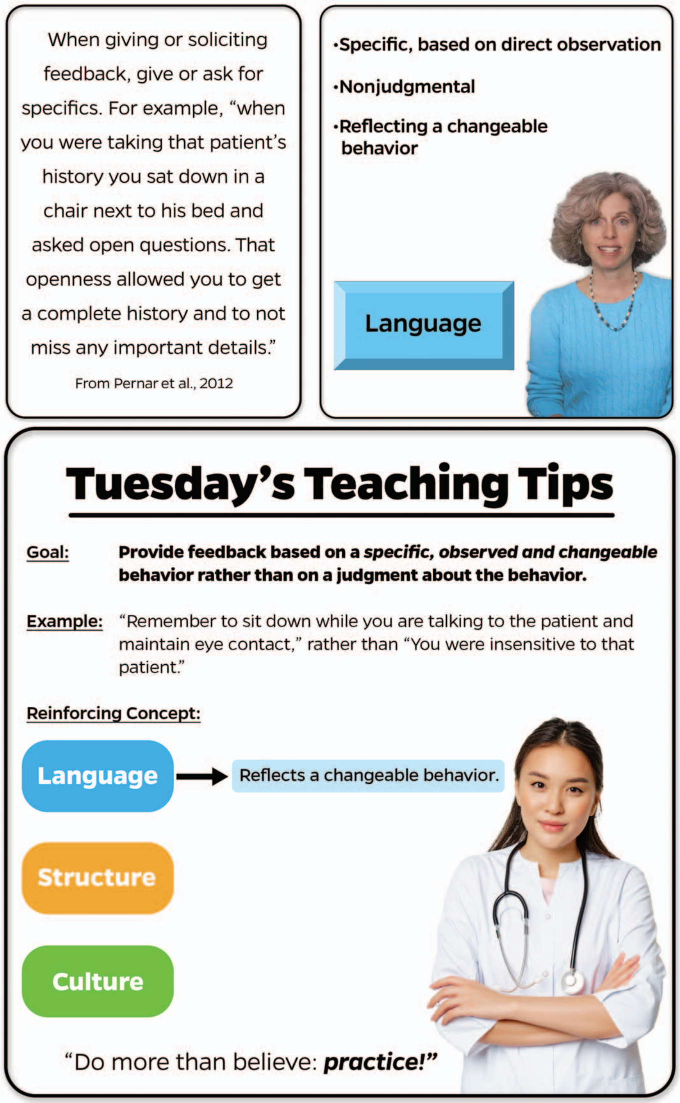

Figure. Deconstructing a Tuesday's Teaching Tip: The top left panel features only a statement (words alone) versus the top right and bottom panels (using CTML solutions) as they appear in the microlecture and tip emails, respectively. The microlecture has limited words in order to focus the learner on the narration and utilizes visual cues (signaling) such as color, bolding, and borders to carry into the tip emails with their additional CTML solutions: arrows, underlining, and aligning. Abbreviation: CTML, cognitive theory of multimedia learning. Top right panel image: author created and owned; bottom panel image: public domain, created by benzoix, https://www.freepik.com/free-photo/portrait-asian-doctor-woman-cross-arms-standing-medical-uniform-stethoscope-smiling-cam_21129522.htm#query=asian%20nurse&position=21&from_view=keyword

present in the microlecture as well as in the emails to assist with memory retrieval, along with simple text (words) and examples to reinforce the content and practical implementation in the clinical learning environment. Utilization of visual cues or signaling is one solution reinforced by CTML to reduce cognitive load in learning. Other solutions include off-loading, segmenting, pretraining, weeding, aligning, eliminating, synchronizing, and

Copyright © 2022 Kalynych et al. This is an open-access publication distributed under the terms of the Creative Commons Attribution license.

individualizing. Table 1 highlights the additional solutions we utilized to reduce cognitive load as part of multimedia learning in the microlecture and emailed tips in order to focus the learner on important content. Furthermore, our emailed tips stressed the importance of practice with trainees, and the program was shorter in duration than in the Matzie and Pernar studies (14 weeks vs. 9 months) to focus the knowledge and practice the skills.

Methods

Curricular Context and Development

We implemented TTT as an optional 14-week faculty development program on evaluation and feedback for teaching physicians across all specialties at one academic institution between February and May 2020. We utilized the framework of spaced education to first provide a foundational microlecture on evaluation and feedback and then reinforce the content through weekly emails containing skills-based tips. The microlecture and emails utilized CTML instructional design solutions to combat cognitive load, which could interfere with multimedia learning. One clinical/medical educator expert and one EdD revised core evaluation and feedback statements and examples from the Matzie and Pernar studies.[23,24] The EdD also designed the microlecture and emails utilizing CTML solutions. The microlecture (Appendix A) was recorded and made available on our institutional website for easy access by participants. Through our email system (Outlook), we emailed small pieces of reinforcing information as weekly statements (Appendix B) that were phrased as skills-based tips for faculty to implement in the clinical learning environment with trainees (students, residents, and/or fellows). Program administrators needed access to a computer, and participants used a computer, tablet, or smartphone to watch the microlecture video and read the weekly emails. We applied for CME credit through our institution, with the 15-minute microlecture as 0.25 hours and each weekly tip and practice as 0.5 hours, for a total of 7.25 CME credits for complete participation. We emailed all the teaching faculty at our institution with a program announcement and directions to enroll (Appendix C). Finally, we utilized Google Forms for faculty to register for the program electronically (Appendix D).

Implementation

The program directions (Appendix E) described the step-by-step implementation process. To those who registered, we sent a preparatory email with overall course instructions and requirements (Appendix F). We utilized our office email account, which was accessible by several members of our staff, and created a distribution list with participants' email addresses. Every Monday afternoon, we set up the tips email for the next day to be sent automatically at 7 a.m. with a read receipt request. We tracked attendance in an Excel spreadsheet using the email read receipts and added ourselves to the email distribution list to ensure that the emails were delivered each Tuesday. We completed several dry runs prior to the start of the course, ensuring that the email system and read receipts worked. Faculty were directed to email our office to document attendance if they encountered issues with their read receipt. The program was run by one EdD and one administrative staff member, requiring no more than 2.5 hours per week for preproduction to recruit, develop the enrollment list and distribution list, and prepare the tip email in advance. During the program, we spent 2 hours per week sending the emails, ensuring delivery, and tracking attendance through read receipts. The administrator spent 3 hours per week postprogram to download evaluations, deidentify evaluations, determine CMEs, and administer certificates. The EdD required 1 hour per week for postproduction, which lasted approximately 3 weeks, to troubleshoot and answer questions.

Table 1. CTML Solutions Utilized

CTML Solutions	Venue	Explanation of Use
Off-loading: Relieve the visual channel by enhancing verbal channel.	Microlecture	We utilized limited words (reading) in the video microlecture (visual channel) and focused on narration, reducing the cognitive load on the visual channel.
Segmenting (time between lessons): Knowledge is transferred better in learner-controlled segments versus continuously.	Spaced education emails	Although we sent out an email each Tuesday and required the participants to read it, the email could be read at anytime before the next Tuesday and implemented at the convenience of the participants and their clinical learning environment.
Pretraining: Provide essential content in advance.	Microlecture	All participants were required to complete the microlecture for foundational information.
Signaling: Institute the use of cues.	Microlecture and spaced education emails	We used visual cues such as colors, highlighting, borders, arrows, and bolding to guide learners to key words or key words in speech or to organize images (mapping). The visual cues were consistent within the microlecture and spaced emails. Additionally, each email was organized the same: goal, example, reinforcing concept.
Aligning words and pictures: Words are placed near graphics to reduce visual scanning (to focus on essential processing).	Microlecture and spaced education emails	See the Figure for an example of the words being placed near graphics for both the microlecture and spaced education emails.

Abbreviation: CTML, cognitive theory of multimedia learning.

Copyright © 2022 Kalynych et al. This is an open-access publication distributed under the terms of the Creative Commons Attribution license.

Program Timeline

- Week 1: We assigned participants to watch the 15-minute microlecture on evaluation and feedback and take the quiz, and we also presented the first tip.
- Weeks 2-13: We emailed one new tip each Tuesday morning, with instructions for participants to intentionally practice the tip with learners during the week.
- Week 14: We sent the program evaluation link to participants. The evaluation results were deidentified by our administrative assistant and given to the TTT program directors. We sent a certificate of completion (Appendix G) to those who had participated, attested to practicing the tips with trainees in at least 11 out of the 13 weeks of TTT, and completed the course evaluation and self-reflection. We notified participants who had completed the microlecture but were unable to participate in at least 11 weeks of the course that they were able to claim CME credit for the weeks they had participated and reported their participation to our CME office.

Evaluation

Our program evaluation plan consisted of utilizing the new world Kirkpatrick model (NWKM) levels 1-3 to analyze data collected from partcipants.[30] Data collection included (1) a short postmicrolecture quiz (Appendix H) at the beginning of the program, which fit into level 2 of the NWKM, and (2) a postprogram evaluation (Appendix I) consisting of required CME questions, a self-reflection question, and an attestation to having completed the practice sessions, which fit into levels 1-3 of the NWKM. Additionally, since this program was implemented just prior to the national shutdown due to COVID-19 in March 2020, we asked if the pandemic had affected respondents' ability to participate. To assess the program objectives, we utilized the microlecture posttest quiz to determine if participants were able to define evaluation and recognize observation as the cornerstone of evaluation (NWKM level 2, learning: knowledge). The postmicrolecture quiz was given electronically through the institutional CME office. The postprogram evaluation was developed in Google Forms by program administrators and administered electronically to participants. We used descriptive statistics to analyze the data into counts and percentages and placed reflective statements into broad categories of similar characteristics for analysis.

Results

Fifty-two physicians across 13 specialties received partial or full CME credit, with 34 (65%) completing the entire program and receiving a course certificate. The 52 physician participants included two from anesthesiology, seven from emergency medicine, one from family practice, two from internal medicine, three from neurology, two from OB/GYN, two from ophthalmology, four from orthopedics, 13 from pediatrics, four from psychiatry, three from radiology, seven from surgery, and two from urology. Forty-eight percent were female. Of the 34 who completed the program, there were 11 different specialties represented, including one participant from anesthesiology, four from emergency medicine, one from family practice, two from internal medicine, two from neurology, one from OB/GYN, four from orthopedics, 11 from pediatrics, one from radiology, five from surgery, and two from urology, with 53% being female.

On the microlecture posttest quiz assessing whether participants were able to define evaluation and recognize observation as the cornerstone of evaluation, 41 of the 52 participants (79%) answered all questions correctly. On the course evaluation, 51 (98%) rated the program as good (n = 12, 23%) or excellent (n = 39, 75%; NWKM level 1, reaction: customer satisfaction), 51 (98%) reported information gained would enhance patient care or medical education (NWKM level 1, reaction: relevance), 51 (98%) had high (n = 40, 77%) or moderate (n = 11, 21%) confidence in their ability to implement changes in their teaching (NWKM level 2, learning: confidence), and 52 (100%) stated they would like to see more TTT on other topics. On the question whether the COVID pandemic had affected respondents' ability to participate, 21 (40%) answered either yes (n = 7, 13%) or somewhat (n = 14, 27%). Of those who answered a follow-up question on the ways the COVID pandemic had affected participation, the majority noted reduced contact with trainees due to canceled educational experiences or virtual interactions with trainees. One respondent had gone on military deployment, and another expressed experiencing burnout. When asked for recommendations for improvement, one participant recommended adding videos or simulated demonstrations, and two participants responded they would have liked a small, laminated card with salient teaching points as a quick reference.

To further assess the ability to describe methods of evaluation and list characteristics of effective feedback, participants who had completed the entire program and received a course certificate (n = 34) were asked to reflect on the course by answering the question "Please describe how this program has improved your overall knowledge and skills related to evaluation and feedback when working with your trainees," with a 500-word limit. Table 2 lists (a) specific characteristics of effective feedback that were identified by 32 (94%) of the 34 participants

Copyright © 2022 Kalynych et al. This is an open-access publication distributed under the terms of the Creative Commons Attribution license.

Table 2. Identified Characteristics of Effective Feedback and Methods of Evaluation

Item	No. of Participants Responding
15 characteristics of effective feedback[a]	
Make feedback specific	15
Based on direct observation	6
Nonjudgmental	4
Reflecting a changeable behavior	5
W questions	8
Feedback sandwiches (old, new)	14
Feedback as an expectation	0
Encourage learner to ask for feedback	0
Limit to 1-2 items	5
Make feedback timely	12
Label it as feedback	7
Use *and* instead of *but*	8
Frequent feedback	2
Action plan	4
Encourage feedback from learner to teacher	8
Method of evaluation[b]	
Ask learner questions	0
Directly observe learner	5
Encourage learner to self-assess (W questions)	7
Will information gained from this program result in enhancing optimal patient care and/or medical education?[c]	52
Yes	51
No	1

[a]Thirty-two of 34 participants (94%) identified at least one characteristic of an effective feedback technique. Participants could choose more than one characteristic. Thirteen of the 15 characteristics (87%) were identified by participants.
[b]Participants could choose more than one method of evaluation.
[c]Fifty-two participants answered this question.

and (b) methods of evaluation in which 13 (87%) of those 15 effective feedback characteristics were identified as being used (NWKM level 3, behavior: critical behaviors). The characteristics of effective feedback most often identified were “make feedback specific,” “utilize a feedback sandwich,” “make feedback timely,” “use the W questions” for learner self-reflection, and “use *and* instead of *but.*”

When reviewing the reflective comments, we grouped statements into two broad categories that appeared to be logical. Many comments concerned (1) the format of TTT and (2) the perceived knowledge and/or skills gained. We identified subgroupings within these two categories. Subgroupings for the format category included the following: (1) The course format was useful for learning feedback, (2) the series of tips helped participants use the tips in busy clinical environments, and (3) participants found it helpful to learn one tip per week. Subgroupings for the knowledge/skills gained category included participants (1) citing specific techniques and principles they had learned and (2) seeing themselves as more effective providers of feedback after the course (Table 3). Participants’ reflective comments assisted with program evaluation at NWKM levels 1 (reaction: customer satisfaction, engagement, and relevance) and 2 (learning: knowledge and skills, attitude and engagement).

Discussion

We developed and implemented an innovative spaced education faculty development program on evaluation and feedback that provided weekly emailed tips utilizing CTML solutions to reduce cognitive load and focus the learners on salient content. Unlike the Pernar study,[24] which found that faculty did not perceive the emailed tips as helpful, our program evaluation found that participants had high satisfaction with the format and were able to identify specific techniques for effective feedback with trainees. Faculty were very receptive to this teaching strategy, as it was time efficient and easily accessible, eliminated the need to go to a training, and offered simple skills-based strategies to practice in the clinical learning environment. Furthermore, 100% of participants reported wanting more TTT instructional sessions in the future as a method for faculty development. It is possible that our use of a foundational microlecture on which to build the spaced education reinforcement and/or the incorporation of CTML solutions encouraged faculty to practice the tips, increased their knowledge and skills, and led to perceived improvements. Additionally, our program was well received by physicians across multiple specialties and was completed over a shorter period of time than the program described in the Matzie and Pernar studies (14 weeks vs. 9 months).[23,24]

Lessons Learned

Given the reliance on technology, being proactive was important to ensuring a smooth implementation of the program. Setting up the tip emails in the email system in advance allowed them to be sent at the designated time each week (Tuesday at 7 a.m.). Practice runs were instrumental in discovering technical glitches with the process, such as with our use of Outlook and our network system. We discovered that if our network system rebooted overnight, it erased emails set up in advance; therefore, we had to ensure each Tuesday morning that the emails had indeed been sent. Moreover, we felt an introductory email describing how the program was designed was imperative to set expectations for the program. Second, we learned to be creative in terms of calculating the time participants would spend on this nontraditional educational activity to earn CME credit. We took into consideration the 15-minute microlecture and determined that to read the email (maybe more than once), practice the tip on one’s own, and then utilize the tip with various learners and at different times during the week, 30 minutes each week was reasonable. We also allowed for partial credit if the microlecture

Copyright © 2022 Kalynych et al. This is an open-access publication distributed under the terms of the Creative Commons Attribution license.

Table 3. Categorized Examples of Comments Relating to Course Format and Perceived Knowledge/Skills Gained

Comment Category	Example Comments
Course Format: Course format was useful for learning feedback.	"Great format."<br>"This course was beneficial, as it provided a well thought out overview of some teaching techniques. Having the goal and then providing the example along [with] the reinforcing concept made it easy for me to contextualize the information."<br>"The course material provided a wonderful structural format to give necessary feedback in a constructive manner while being in an appropriate environment."<br>"This was a wonderful program, and I recognized from the tips and lectures that my feedback to the residents was probably antiquated."
Course Format: Series of tips helped participants use the tips in busy clinical environments.	"Tips were very practical and also easy to implement even in a busy chaotic workplace."<br>"The design of this course also allowed me to easily incorporate it into my workflow and my various competing demands."<br>"This program has allowed me to realize that productive interaction between teacher and learner can be achieved in small increments of time which can be completed in clinical space without interruption of workflow."<br>"In particular, the regular, short tips with specific guides to practice the techniques provided me with the chance to participate in the activity despite challenges in scheduling that often preclude such opportunities."
Course Format: Participants found it helpful to learn one tip per week.	"Having each of the individual tips rolled out weekly allowed me to focus on each activity and see how I can seamlessly incorporate it into my own style of teaching. As the weeks progressed and new tips became available, I found it helpful to incorporate the new activities/behaviors and layer them on top of the old ones."<br>"These concise lessons allowed [me] to focus [on] one area of improvement every week. The course clearly demonstrated how each of the reinforcing concepts are dependent on each other."<br>"This was a great format for this type of learning. I enjoyed the specific advice weekly and plan to work on implementing this into my practice as a junior faculty member."<br>"I would like to see more of these type of weekly tips, as they are helpful without being a chore like some other CME formats can be."
Perceived Knowledge/Skills Gained: Participants cited specific techniques and principles they learned.	"I was able to be more specific, making a point during my feedback [of] avoiding judgement.… I also used the W questions."<br>"Reflecting… on feedback, I felt I have changed the way I have discussions with residents.… I have switched to the ask, tell, ask model of feedback.… I have also made a conscious effort to give feedback to the residents I work with as soon as possible."<br>"The program significantly improved my skills in teaching medical students and residents.… I am now able to give brief and timely feedback, be more observant and give specific feedback based on direct observation."<br>"I will continue trying to work on more timely and specific feedback; using 'and' versus 'but' certainly made a difference, it didn't feel like I'm telling somebody they did something wrong or they asked a wrong question."
Perceived Knowledge/Skills Gained: Participants saw themselves as more effective providers of feedback after the course.	"Tuesday's Teaching Tips have improved my overall knowledge and skills related to evaluation and feedback when working with my trainees."<br>"The knowledge gained through Tuesday's Teaching Tips has helped me become a more effective educator by enhancing my feedback skills."<br>"After completing this course, I am confident in providing appropriate feedback to the residents and fellows in a structured manner."<br>"The program significantly improved my skills in teaching medical students and residents, even my medical assistants."

had been completed. Even though faculty signed up for the program, if they did not watch the microlecture as foundational information, they did not receive the TTT emails (the spaced education).

Limitations

Our evaluation was based on participants' self-report and not on observations of their teaching or resident evaluation of their teaching before and after implementation of the program (level 4 of NWKM) for educational outcomes. In addition, our evaluation approach was somewhat limited by the use of our institutional CME system. The quiz after the microlecture did not accurately capture correct and incorrect questions individually but rather a pass/fail; therefore, we included only 100% passing scores to assess objectives 1 and 2. There could have been more correct answers for each question if we had individual question data. For the postprogram evaluation, we utilized Google Forms to include the required CME questions and were able to add the self-reflection question and attestation. We were mindful of keeping the evaluation feasible for busy clinicians. Satisfaction questions were based on all 52 participants who received CME credit as all 52 completed the course evaluation. However, only those who both completed the course and earned the course certificate received the self-reflection questions. We did not assess participants' prior experience with or training in evaluation and feedback, which may have impacted the interpretation of the program evaluation results. Finally, we did not do a true qualitative thematic analysis of the reflective comments but rather grouped them into broad categories.

Copyright © 2022 Kalynych et al. This is an open-access publication distributed under the terms of the Creative Commons Attribution license.

TTT is an approach that addresses faculty development barriers of faculty time constraints, convenience in attending, and perceived benefits. The program was easy to implement, required minimal staff time, and used the Outlook email system for program delivery as well as an Excel spreadsheet to track participation. The program has broad applicability across multiple medical specialties in an academic medical center with no obvious challenges to generalizability. The tips can be integrated into an existing faculty development curriculum or utilized as a stand-alone program. Next steps involve a qualitative analysis of the participants' reflective statements. Future research or evaluation should include the behavioral impact of TTT on faculty skills as well as the impact of TTT on learners.

Appendices

A. Evaluation and Feedback Microlecture.m4v
B. Emailed Tips.pptx
C. Program Announcement.pptx
D. Registration Form.docx
E. Program Directions.docx
F. Preparatory Email.docx
G. Certificate of Completion.docx
H. Postmicrolecture Quiz.docx
I. Postprogram Evaluation.docx

All appendices are peer reviewed as integral parts of the Original Publication.

Colleen Kalynych, EdD: Assistant Dean for Medical Education, Office of Educational Affairs, and Senior Lecturer, Department of Emergency Medicine, University of Florida College of Medicine–Jacksonville

Linda Edwards, MD: Dean and Associate Professor of Medicine, University of Florida College of Medicine–Jacksonville

Denise West, MA: Assistant Director, Office of Educational Affairs, University of Florida College of Medicine–Jacksonville

Charity Snodgrass: Administrative Support, Office of Educational Affairs, University of Florida College of Medicine–Jacksonville

Elisa Zenni, MD: Senior Associate Dean for Educational Affairs, Office of Educational Affairs, and Professor of Pediatrics, Department of Pediatrics, University of Florida College of Medicine–Jacksonville

Disclosures
None to report.

Funding/Support
None to report.

Prior Presentations
Kalynych CJ, Edwards L, West D, Snodgrass C, Zenni E. Tuesdays are great for teaching tips: a spaced education strategy for faculty development. Poster presented virtually at: 2021 AAMC Group on Educational Affairs Spring Regional Meeting; April 20-22, 2021.

Kalynych CJ, Sottile E, Edwards L, Snodgrass C, Zenni E. Tuesdays Teaching Tips: a spaced education strategy for faculty development. Poster presented virtually at: 2022 ACGME Annual Educational Conference Virtual Experience; March 30-April 1, 2022.

Ethical Approval
Reported as not applicable.

References

1. *ACGME Common Program Requirements (Residency).* Accreditation Council for Graduate Medical Education; 2020. Accessed September 19, 2022. https://www.acgme.org/Portals/0/PFAssets/ProgramRequirements/CPRResidency2020.pdf
2. Khan N, Khan MS, Dasgupta P, Ahmed K. The surgeon as educator: fundamentals of faculty training in surgical specialties. *BJU Int*. 2013;111(1):171-178. https://doi.org/10.1111/j.1464-410X.2012.11336.x
3. McLean M, Cilliers F, Van Wyk JM. Faculty development: yesterday, today and tomorrow. *Med Teach*. 2008;30(6):555-584. https://doi.org/10.1080/01421590802109834
4. Ramani S, Leinster S. AMEE Guide no. 34: teaching in the clinical environment. *Med Teach.* 2008;30(4):347-364. https://doi.org/10.1080/01421590802061613
5. Skeff KM, Stratos GA, Mygdal W, et al. Faculty development: a resource for clinical teachers. *J Gen Intern Med.* 1997;12(suppl 2):S56-S63. https://doi.org/10.1046/j.1525-1497.12.s2.8.x
6. Steinert Y, Macdonald ME, Boillat M, et al. Faculty development: if you build it, they will come. *Med Educ*. 2010;44(9):900-907. https://doi.org/10.1111/j.1365-2923.2010.03746.x
7. Carraccio C, Wolfsthal SD, Englander R, Ferentz K, Martin C. Shifting paradigms: from Flexner to competencies. *Acad Med.* 2002;77(5):361-367. https://doi.org/10.1097/00001888-200205000-00003
8. Epstein RM. Assessment in medical education. *N Engl J Med.* 2007;356(4):387-396. https://doi.org/10.1056/NEJMra054784
9. Holmboe ES, Sherbino J, Long DM, Swing SR, Frank JR; International CBME Collaborators. The role of assessment in competency-based medical education. *Med Teach*. 2010; 32(8):676-682. https://doi.org/10.3109/0142159X.2010.500704
10. Bing-You RG, Trowbridge RL. Why medical educators may be failing at feedback. *JAMA*. 2009;302(12):1330-1331. https://doi.org/10.1001/jama.2009.1393
11. Blankush JM, Shah BJ, Barnett SH, et al. What are the associations between the quantity of faculty evaluations and

Copyright © 2022 Kalynych et al. This is an open-access publication distributed under the terms of the Creative Commons Attribution license.

residents' perception of quality feedback? *Ann Med Surg (Lond)*. 2017;16:40-43. https://doi.org/10.1016/j.amsu.2017.03.001

12. LaDonna KA, Hatala R, Lingard L, Voyer S, Watling C. Staging a performance: learners' perceptions about direct observation during residency. *Med Educ.* 2017;51(5):498-510. https://doi.org/10.1111/medu.13232
13. Massie J, Ali JM. Workplace-based assessment: a review of user perceptions and strategies to address the identified shortcomings. *Adv Health Sci Educ Theory Pract.* 2016;21(2): 455-473. https://doi.org/10.1007/s10459-015-9614-0
14. Veloski J, Boex JR, Grasberger MJ, Evans A, Wolfson DB. Systematic review of the literature on assessment, feedback and physicians' clinical performance: BEME Guide no. 7. *Med Teach.* 2006;28(2):117-128. https://doi.org/10.1080/01421590600622665
15. Young JQ, Sugarman R, Schwartz J, O'Sullivan PS. Overcoming the challenges of direct observation and feedback programs: a qualitative exploration of resident and faculty experiences. *Teach Learn Med.* 2020;32(5):541-551. https://doi.org/10.1080/10401334.2020.1767107
16. Ende J. Feedback in clinical medical education. *JAMA*. 1983;250(6):777-781. https://doi.org/10.1001/jama.1983.03340060055026
17. Hernandez MR, Wang JT. Feedback in medical education is a journey; pack more than a sandwich. *Update Anaesth*. 2022;36:41-46. https://resources.wfsahq.org/wp-content/uploads/Update-35-Anaes_In_Med_Ed_WFSA_D_21_00008b.pdf
18. Holmboe ES, Ward DS, Reznick RK, et al. Faculty development in assessment: the missing link in competency-based medical education. *Acad Med*. 2011;86(4):460-467. https://doi.org/10.1097/ACM.0b013e31820cb2a7
19. Jeremiah J, McGarry K, Monteiro JF, Tammaro D. How to build it so they'll come: faculty opinion on faculty development. *R I Med J (2013)*. 2021;104(3):58-61.
20. Kim B, Rajagopalan A, Tabasky EM, Reddy SS, Topor DR. Sharing perspectives on feedback: a combined resident-faculty workshop. *BMC Med Educ*. 2022;22:197. https://doi.org/10.1186/s12909-022-03253-6
21. Kogan JR, Conforti LN, Bernabeo EC, Durning SJ, Hauer KE, Holmboe ES. Faculty staff perceptions of feedback to residents after direct observation of clinical skills. *Med Educ.* 2012;46(2):201-215. https://doi.org/10.1111/j.1365-2923.2011.04137.x
22. Lockyer J, Carraccio C, Chan M, et al.; ICBME Collaborators. Core principles of assessment in competency-based medical education. *Med Teach.* 2017;39(6):609-616. https://doi.org/10.1080/0142159X.2017.1315082
23. Matzie KA, Kerfoot BP, Hafler JP, Breen EM. Spaced education improves the feedback that surgical residents give to medical students: a randomized trial. *Am J Surg.* 2009;197(2):252-257. https://doi.org/10.1016/j.amjsurg.2008.01.025
24. Pernar LIM, Beleniski F, Rosen H, Lipsitz S, Hafler J, Breen E. Spaced education faculty development may not improve faculty teaching performance ratings in a surgery department. *J Surg Educ.* 2012;69(1):52-57. https://doi.org/10.1016/j.jsurg.2011.06.013
25. Kang SHK. Spaced repetition promotes efficient and effective learning: policy implications for instruction. *Policy Insights Behav Brain Sci*. 2016;3(1):12-19. https://doi.org/10.1177/2372732215624708
26. Mayer RE, Moreno R. Nine ways to reduce cognitive load in multimedia learning. *Educ Psychol*. 2003;38(1):43-52. https://doi.org/10.1207/S15326985EP3801_6
27. Mayer RE. Applying the science of learning to medical education. *Med Educ*. 2010;44(6):543-549. https://doi.org/10.1111/j.1365-2923.2010.03624.x
28. Sweller J, van Merrienboer JJG, Paas FGWC. Cognitive architecture and instructional design. *Educ Psychol Rev*. 1998;10(3):251-296. https://doi.org/10.1023/A:1022193728205
29. Young JQ, Van Merrienboer J, Durning S, Ten Cate O. Cognitive load theory: implications for medical education: AMEE Guide no. 86. *Med Teach*. 2014;36(5):371-384. https://doi.org/10.3109/0142159X.2014.889290
30. Kirkpatrick J, Kirkpatrick WK. *An Introduction to the New World Kirkpatrick Model*. Kirkpatrick Partners; 2021. Accessed September 19, 2022. https://www.kirkpatrickpartners.com/wp-content/uploads/2021/11/Introduction-to-the-Kirkpatrick-New-World-Model.pdf

Received: April 22, 2022
Accepted: August 19, 2022
Published: November 22, 2022

Copyright © 2022 Kalynych et al. This is an open-access publication distributed under the terms of the Creative Commons Attribution license.
